# Skill learning and the evolution of social learning mechanisms

**DOI:** 10.1186/s12862-016-0742-9

**Published:** 2016-08-24

**Authors:** Daniel J. van der Post, Mathias Franz, Kevin N. Laland

**Affiliations:** 1 Center for Social Learning and Cognitive Evolution, School of Biology, St Andrews University, Harold Mitchell Building, St Andrews, KY16 9TH UK; 2 Leibniz Institute for Zoo and Wildlife Research, Alfred-Kowalke-Straße 17, Berlin, 10315 Germany

**Keywords:** Multi-scale approach, Agent-based model, Information parasitism, Mechanism specificity, Group foragers, Self-organization

## Abstract

**Background:**

Social learning is potentially advantageous, but evolutionary theory predicts that (i) its benefits may be self-limiting because social learning can lead to information parasitism, and (ii) these limitations can be mitigated via forms of selective copying. However, these findings arise from a functional approach in which learning mechanisms are not specified, and which assumes that social learning avoids the costs of asocial learning but does not produce information about the environment. Whether these findings generalize to all kinds of social learning remains to be established. Using a detailed multi-scale evolutionary model, we investigate the payoffs and information production processes of specific social learning mechanisms (including local enhancement, stimulus enhancement and observational learning) and their evolutionary consequences in the context of skill learning in foraging groups.

**Results:**

We find that local enhancement does not benefit foraging success, but could evolve as a side-effect of grouping. In contrast, stimulus enhancement and observational learning can be beneficial across a wide range of environmental conditions because they generate opportunities for new learning outcomes.

**Conclusions:**

In contrast to much existing theory, we find that the functional outcomes of social learning are mechanism specific. Social learning nearly always produces information about the environment, and does not always avoid the costs of asocial learning or support information parasitism. Our study supports work emphasizing the value of incorporating mechanistic detail in functional analyses.

**Electronic supplementary material:**

The online version of this article (doi:10.1186/s12862-016-0742-9) contains supplementary material, which is available to authorized users.

## Background

Social learning is of considerable interest as both a means of behavioral adaptation and a prerequisite for cultural inheritance [1, 2]. Researchers have hypothesized that particular social learning mechanisms promote complex culture because those mechanisms generate sufficiently high-fidelity copying to allow culture to increase in complexity across generations [3–12]. Social learning mechanisms would therefore appear to play a critical role in the evolution of complex culture.

The term ‘social learning mechanism’ refers largely to the kinds of cues that individuals pay attention to when learning from conspecifics [2]. For instance, ‘local enhancement’ and ‘stimulus enhancement’, respectively, prime individuals to approach particular locations, or to interact with particular kinds of objects in the environment, because other individuals have been observed to be in those locations or to have interacted with those objects [2]. In contrast, ‘production imitation’ [13] leads individuals to copy a process or method of interacting with a particular object. There now exists considerable empirical support for the existence of these and several other social learning mechanisms [2].

In spite of this established mechanistic variation, most theory on the evolution of social learning does not consider mechanisms explicitly. Instead, the aim is to represent social learning in a general way. Behavior and learning are therefore specified on a functional level, for instance in terms of payoffs in a game-theoretic fashion [14], with social and asocial learning typically compared as alternative learning strategies [15–20]. To do so, modelers make assumptions about the payoffs and information that are generated by social and asocial learning. Innovation and asocial learning are assumed to produce information about the environment, but at a cost [15]. In contrast, social learning is typically implemented as a strategy that ‘avoids the costs’ of asocial learning by copying existing behavior, but does not produce information about the environment. This ‘avoiding costs’ scenario makes sense given that social learning may increase the rate of learning (alleviate time costs), lead to less energy expenditure (alleviate energy costs), and/or avoid risks such as poisoning (reduce risk costs).

The payoffs and information production of the above models, lead to an important result, namely ‘information parasitism’. There is an incentive to copy, because social learning enables the costs of asocial learning to be avoided. However, there is a catch: in such formulations social learning does not generate new information about the environment. In changing environments, negative frequency-dependence is therefore generated, whereby the random (non-selective) social learning deployed is only advantageous when rare, and at the mixed equilibrium social learning does not increase average fitness in the population [15]. This outcome is known as Rogers’ paradox, and has led to the expectation that social learning should be selective, with respect to when, from whom, or what, individuals copy [2, 6, 14, 21, 22]. The success of this theory is reflected in the fact that the trade-off between social and asocial learning, and resultant information parasitism, is now a common expectation with respect to the evolution of social learning in general [2].

However, the above summarized theory is subject to limitations. First, as mechanisms are not specified, the theory cannot be used to understand how different social learning mechanisms, and resultant cultural phenomena, evolve. Secondly, given the neglect of mechanism, it is difficult to know if the predictions of the game-theoretic style models are truly general, or if they result from specific, and possibly biologically unlikely, assumptions of the model architecture such as assumptions about payoffs and information production.

These concerns are reinforced by recent theoretical analyses which show that consideration of behavioral mechanisms can make a difference to functional outcomes, or payoffs, in evolutionary analyses [23–26]. These findings suggest caution is warranted when generalizing across the various social learning mechanisms. Moreover, there are specific reasons for thinking that social learning need not lead to information parasitism [27]. Models with mechanistic detail provide ‘proofs of principle’ revealing that social influences can occur during the learning process so that social and personal information are integrated, rather than trading-off against each other [1, 27–30]. Thus, as suggested by definitions of local and stimulus enhancement and empirical evidence thereof [2], much ‘social learning’ is actually well-characterized as socially guided individual learning, which means that the use of social information need not imply that the learner has not monitored the state of the environment. Such findings suggest that instead of generating information parasitism, social influences can under some circumstances enhance information production, generating opportunities for new learning outcomes [27, 31], most obviously where social learning supports cumulative cultural learning [32].

It is possible to study how social learning enhances information production in game-theoretic models, but this requires special strategies that determine when individuals should copy others [20, 33, 34]. Conversely, in detailed mechanistic models, such outcomes are possible without special strategies [27, 31, 35], raising the possibility that the model architecture in the game-theoretic approach may overemphasize the importance of complex decision rules, and even simple strategic biases. To date, however, mechanistic models have not tended to incorporate evolution. It therefore remains an open question whether, and in what socio-ecological contexts, individuals that learn socially will explore or innovate less than individuals that learn asocially due to the effects of information parasitism, and whether special social learning strategies are required to generate opportunities for new learning outcomes.

Here, we aim to examine how different social learning mechanisms relate to information production and payoffs, and how information production affects the evolution of mechanisms. Given the aforementioned game-theoretic predictions, we assume that any form of social learning could in principle avoid costs and generate information parasitism. However, in our model we do not predefine whether social learning enables the costs of asocial learning to be avoided, nor whether social learning will trade-off with information production. Instead, we implement different social learning mechanisms in multi-scale evolutionary models, which allows us to study how payoffs and information production arise. In this approach [24, 36–41], the mechanisms are defined at a local spatio-temporal scale in local socio-ecological contexts in terms of effects of learning during behavioral events, based on existing models of reinforcement learning [42] and definitions of social learning mechanisms [2]. We then study the outcome of learning mechanisms across larger timescales (information production and payoffs), and its effect on lifetime reproductive success (fitness) of individuals. In this way we establish how mechanisms at the local scale map onto information production and payoffs at a socio-ecological scale. The latter correspond to the payoffs and information production that are assumed in the above mentioned game-theoretic style models. Our approach therefore enables us to assess whether the evolution of the local mechanisms leads to the evolution of the payoffs and information production that are commonly assumed in the above game-theoretic models.

We build on previous multi-scale theory on group foraging where ‘learning what to eat’, ‘the evolution of foraging’ and the ‘evolution of grouping’ have been thoroughly examined in comparable ecological settings [24, 27, 28, 31, 35, 39, 41]. Here we focus on group foragers learning what and how to eat in patchy environments. We consider this a suitable context for primates, which are a relevant taxa for which to consider the evolution of social learning mechanisms and cultural phenomena. We compare three different social learning mechanisms, namely local enhancement, stimulus enhancement and observational learning.

In our model, *local enhancement* arises spontaneously due to grouping, because if individuals approach each other and stay together they automatically affect each other’s learning opportunities [28]. Such coarse-grained local enhancement occurs indirectly as a group-level process. In contrast, *stimulus enhancement* and observational learning are direct results of the specific sensing and decision making of an individual [2, 43]. Here, following van der Post et al. [27], stimulus enhancement is modelled as an increase in the probability that a forager processes and consumes a resource type after observing another forager interacting with that resource type. *Observational learning*, following Franz and Mathews [29], is here represented by an increase in the skill with which a forager processes a specific resource type after observing another forager processing that resource type. This implementation of observational learning includes a potentially large set of social learning mechanisms, including production imitation [2].

Using this model, starting from the baseline of no social learning, we investigate if and how the different kinds of social learning mechanism evolve in group foragers learning what and how to eat. We pursue three goals. First, by manipulating the difficulty of developing processing skills and the rate of environmental change, we aim to gain a greater understanding of the conditions under which different social learning mechanisms are beneficial. Second, we examine whether, and under what circumstances, information parasitism arises, by investigating when the evolution of social learning leads to a corresponding reduction in the exploration rate, and whether that reduction occurs because there is an incentive for foragers to avoid the costs of exploration. Finally, we examine whether, and under what circumstances, social learning can generate opportunities for novel learning outcomes, enabling information production that goes beyond the capabilities of asocial learners.

## Methods

Our model is an event-based, individual-based model with a spatially-explicit environment and is freely available at https://bitbucket.org/dvanderpost/aapjes_bmc_eb_2016. The key design feature of the model is that we define behavioral decision making and the outcome of behavioral events, including learning, at a local spatio-temporal scale. We then study the meso- and macro-scale consequences of that local behavior to establish the mapping between different mechanisms at a local scale and information processing and payoffs at a larger scale. While the model is formulated ‘keeping primates in mind’, and a large number of parameter values are based on estimates of natural primate systems, we expect our conclusions to generalize to other animal taxa, particularly those with similar movement patterns and repertoire sizes. The model is based on previous models of learning in group foragers [27, 28, 31], but now includes skill learning, observational learning, dynamic populations and group sizes, and evolving parameters. The following model description is limited to those aspects needed to gain a reasonable understanding of the results, with key parameters listed in Table 1. For further details see Section 1 in Additional file 1.

**Table 1:** List of key parameters

Name	Description	Values
*R*	Number of resources species	250
*Q*_*r*_	Maximum energy reward of resource	*N*(0.1,0.1)
	type *r*	
*H*_*r*_	Practice time needed before obtaining	
	half the maximal reward	0.1..10
*S*_*r*_	Scalar for the sigmoid function	
	describing how rewards increase	
	with practice	1..4
*EC*	Rate at which resource	
	types are replaced by new types	0..*R* types per year
*N*	Population size	100
*G*	Maximum number of foragers in a group	20
COPY_SPACE	Distance at which foragers	
	can observe what their neighbors	20
	are doing	
*K*	Effectiveness of	
	observational learning	0.1
*λ*_*i*_	Reinforcement learning rate	0..1
*ε*_*i*_	Exploration rate	0..1
*γ*_*i*_	Stimulus enhancement	0..1
*ω*_*i*_	Probability to OBSERVE neighbor	0..1
*τ*_*i*_	Duration of OBSERVE	0.01..1 min

Upper case letters and names: invariant parameters that do not change during simulations. Greek letters: parameters that can evolve but are invariant during a forager’s lifetime. Subscripts: *i*= forager identity; *r*= resource type

### Model overview

We first give a short overview of the model, followed by further details.

**Fig. 1:**
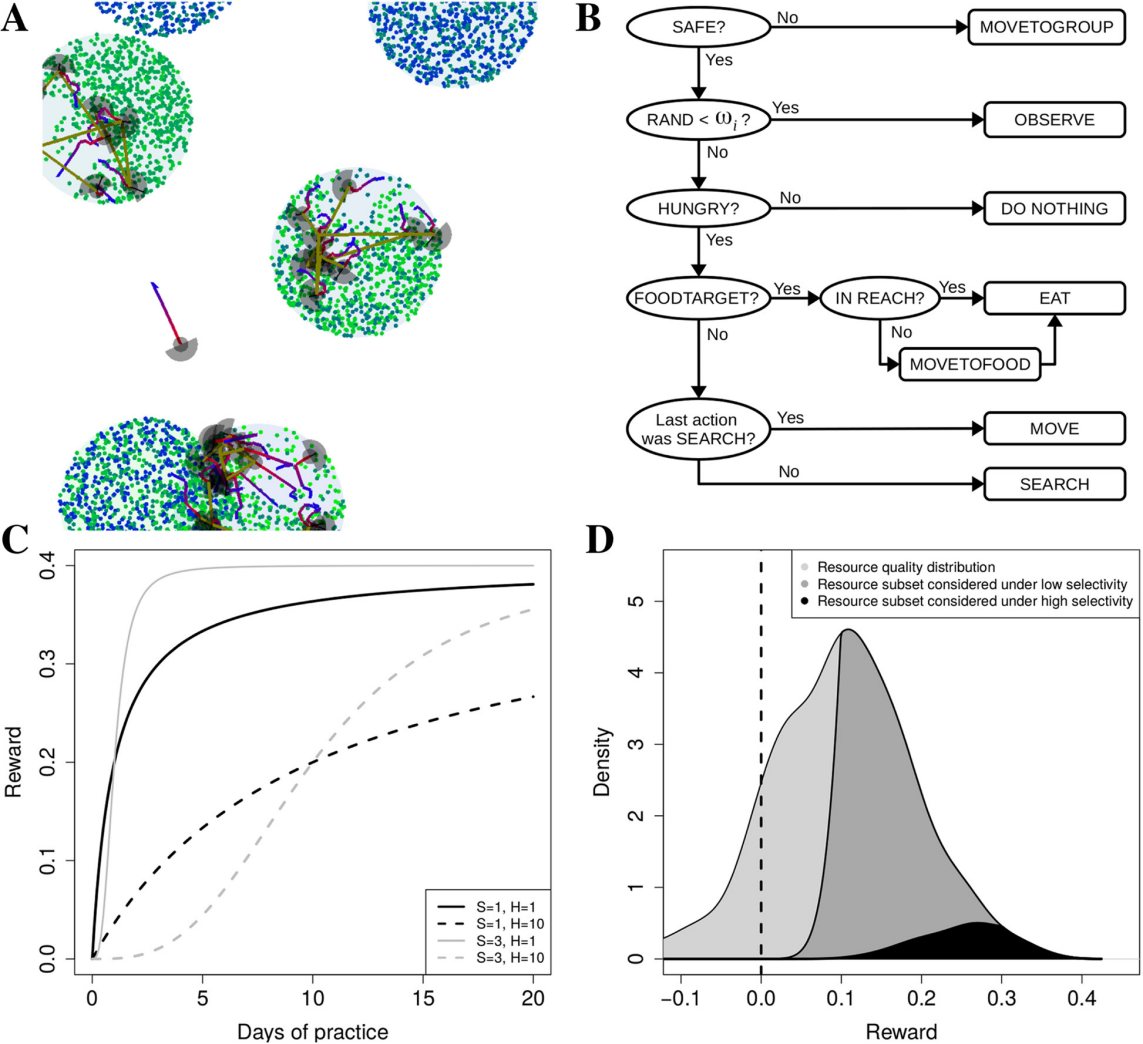
Model details. **a** Simulation snapshot. Each forager is indicated by a SEARCH area (*gray semi-circle*), REACH (gray circle) and a movement trajectory (*red to blue line*). When a foragers observes another forager the foragers are connected by an *olive-green* line. For illustration purposes, the resource items are shown as *colored circles*, and patches by a *larger gray circles*. Each patch can be assumed to be a distinct patch type, with unique resource types (different colours within a patch). **b** Illustration of decision-making algorithm. Rectangles are actions and ellipses are decision-making points. After completing one of the actions at the right hand side, all foragers start the decision-making process at the top left (SAFE?). RAND is a random number between 0 and 1, and *ω*_*i*_ is the probability to do OBSERVE. MOVETOFOOD is always followed by EAT. MOVE consists of at many 1 meter steps to complete a distance of *δ*_*i*_. **c** Illustration of how rewards *e*_*ir*_ change with time spent practicing that skill for different resource types (Eq. 5): resources for which not much practice is needed (*solid lines*, low *H*) and those for which a lot of practice is need (*dashed line*, high *H*); and resources for which rewards increase fast immediately (black lines, low *S*) and those for which they increase slowly initially (*gray lines*, high *S*). **d** Illustration of how selectivity (Eq. 1) affects which subset of resources are chosen: overall resource quality distribution given by *N*(0.1,0.1) (*light gray*) and subsets chosen when selectivity is low (*dark gray*, *a*_*ie*_=0.1) and high (*black*, *a*_*ie*_=0.3), given *σ*_*i*_=5 and assuming the forager knows all resources perfectly

**Entities:** The model is composed of groups of foragers and patches made up of resource items, which are situated in continuous space (Fig. 1a and Additional file 2).

**State variables:** Resources items are defined by a position, and a type which is characterized by quality *Q*_*r*_, and two parameters defining how difficult the resource type is to process (*H*_*r*_ and *S*_*r*_), or ‘task difficulty’. *H*_*r*_ defines the practice time (or experience) needed to develop half of the maximal skill for that resource type, and *S*_*r*_ defines the shape of the function of how skill increases with experience (see ‘Skill learning’ below). Patches are emergent from clumps of resource items in space, and have a type defined by a set of 5 resource types that only occur in patches of that type. Foragers are defined by a position and heading, a current action and a time to its completion, short-term memory about movement and foraging goals, and long-term memory about the rewards associated with resources and resource processing skill. Foragers can differ in their information about resources and skill levels, as well as in their propensity for learning as defined by parameters that can mutate (see Table 1).

**Processes and scheduling:** The implemented processes in our model can be organized hierarchically as: (i) local decision making and movement of foragers; (ii) learning; (iii) life-history updating and demographics; and (iv) environmental updating.

Local decision making is governed by a decision-making algorithm which encodes sensing, decision making, movement, grouping and the updating of short-term memory. In simulations with grouping, foragers belong to a particular group, and follow behavior rules that ensure that groups move cohesively through the environment. All foragers are placed in a queue according to the time their action ends. The forager with the least time remaining is next to choose an action and is put back in the queue according to the time its new action ends. In this event-based setup, actions of foragers can overlap in time, and some foragers can complete multiple quick actions (e.g. move) while others are engaged in actions that take more time (e.g. searching for food).

The learning algorithms include representations of individual and social learning, and update long term memory about properties of resources that foragers interact with as a consequence of their decisions.

Life-history updating occurs at regular time intervals and includes: (i) metabolism or energy expenditure; (ii) digestion of consumed resources; (iii) deaths and (iv) births of foragers; and (v) splitting of groups. After a forager dies, a forager is selected from the remaining population to reproduce, thus maintaining a fixed population size. Foragers are selected to reproduce in relation to their energy levels, where a doubling in energy leads to an 8-fold increase in the probability to reproduce. Offspring inherit the parameter values of their parents with a chance of mutation (see Table 1). In simulations with grouping, groups grow due to births until they reach a maximum size, and then split randomly into two equally sized daughter groups. Groups shrink due to deaths and disappear when the last group member dies.

Environmental updating occurs at regular intervals and involves the ‘growth’ of all resource items at the beginning of each year and ‘environmental change’ that changes an existing resource types into an unknown (for foragers) new resource type. ‘Resource consumption’ occurs when foragers consume resources as determined by ‘local decision making’.

**Spatio-temporal scaling:** The environment is a continuous space of about 40 km^2^, foragers take steps of a meter at a speed of 0.5 m/s, and patches are 20 meters in diameter (Fig. 1a and Additional file 2). Foragers can observe resources up to 2 meters away, and can observe which resources their neighbors are interacting with at 20 meters (a best case scenario for social learning, Additional file 3). There are no constraints on observing group members for grouping purposes in order to ensure cohesive groups, but the spread of groups tends to be in the order of 5–40 meters. All movement occurs in continuous space and there are no constraints on direction.

The timescale is defined in terms of the foragers’ behavioral actions that vary in duration from about a few seconds to a minute. In the model a year is defined as 360 days, and a day is 12 h or 720 min, where we focus on daylight time in a day. Thus foragers can complete many hundreds of behavioral actions in a day and learn from them. Energy expenditure (metabolism) occurs every minute. Digestion occurs every 100 min (DIGESTIONTIME). Foragers can live maximally for 20 years, but can die before that at any minute.

### Resources

In our default setting, resource items of 250 resource types are distributed in 24500 patches with 1200 items each. There are 50 patch types, and a patch type is characterized by the presence of five resources types that only occur in that patch type (as in trees with fruit, leaves, flowers etc). In order to generate variation across patches of a given type, each patch of a given type is defined by three resource types which are randomly selected from the five resource types that characterize that patch type.

While these parameter values typically underestimate the diversity of natural environments, we strike a pragmatic balance between model complexity and simulation environments that are too simple, and where learning hardly plays a role [28]. We compare this ecological context with randomly distributed resources without patches, and pure patches where each patch type has only one resource type.

Resource items disappear when consumed by foragers, and are then unavailable for consumption. Resource ‘growth’ happens once a year, when all resource items that have been consumed by foragers reappear in the exact same position (for computational reasons) and with the same type. Environmental change occurs randomly at any minute with a given probability and changes a randomly selected resource type into another newly generated resource type which is unfamiliar to the foragers. For ease of interpretation we express this as a rate, namely how many resource types change per year (*EC*). All resource items of the disappearing type change into the new resource type. We vary *EC* across simulations to determine the effect of environmental change. We compare this kind of environmental change to one where resources do not disappear and change into new ones, but where resources remain familiar but change in quality.

The quality of a resource type *Q*_*r*_ is drawn from a random distribution with mean 0.1 and standard deviation of 0.1 (Fig. 1b light gray), and all items of a given resource type have the same quality. Thus we generate variation in quality across resource types which enables the learning process to be studied as an optimization process. Quality defines the maximal reward that a forager can obtain from a resource type when it has sufficient experience with processing that resource type. Task difficulty is defined by *H*_*r*_, the practice time (or experience) needed to obtain half of the maximal reward of that resource type, and *S*_*r*_, which defines how the reward increases with experience (see ‘Skill learning’ below). *S*_*r*_ varies randomly between 1 and 4 (integer values only) and *H*_*r*_ is varied across simulations to determine an overall difficulty of learning in the environment.

### Local decision making

Foragers can choose between several local actions, namely, MOVE, SEARCH, MOVETOFOOD, EAT, MOVETOGROUP, OBSERVE and NOTHING, which are selected according to a decision-making algorithm (Fig. 1b). In the algorithm, individuals start by checking if they are safe (CHECKSAFE), which implies having a sufficient number of neighbors (9) in SAFESPACE (17 meters). During CHECKSAFE, foragers can also observe neighbors within COPYSPACE (20 meters), and can monitor the resources with which those neighbors interact (Fig. 1a). These observations are relevant for stimulus enhancement (SE) and observational learning (OL).

If not safe, foragers do MOVETOGROUP, which means that a forager moves towards the center of its group, calculated as the mean position of the other members of its group (Fig. 1b, first line). Once safe, the forager then aligns its own heading with the average direction of other members of its group in ALIGNSPACE (20 meters). This attraction-alignment algorithm ensures that foragers stay together but travel in a relatively efficient manner through the environment.

If safe, foragers do OBSERVE (*τ*_*i*_ minutes) with probability *ω*_*i*_, which leads to observational learning (OL, see below; (Fig. 1b, second line). Otherwise, with probability 1−*ω*_*i*_, foragers will select one of the remaining actions. If foragers are not HUNGRY (stomach content is at a maximum capacity of 20 resource items), foragers will do NOTHING (1 minute; Fig. 1b, third line). Stomach contents are reset to zero at DIGESTIONTIME.

If HUNGRY, and if they have already selected a resource item for consumption (FOODTARGET), foragers will EAT (1 min.), or MOVETOFOOD if the item is beyond reach (0.9 meters) and EAT once the item is within reach (Fig. 1b, fourth line). If foragers do not yet have a FOODTARGET but their last action was SEARCH, this means they did not find any resource items in view sufficiently attractive and then they will MOVE forward *δ*_*i*_ meters in the direct the foragers is facing (Fig. 1b, fifth line). If they did not yet SEARCH, they will SEARCH (Fig. 1b, sixth line). During SEARCH up to 20 resource items in view (2 meters) are assessed in sequence (Fig. 1a, grey semi-circles). The 20 items are randomly selected from those in view. The search terminates as soon as an item is chosen for consumption, or when none of the items is chosen.

### Food choice algorithm

During SEARCH, a forger’s decision to EAT a given resource item is determined by its (i) exploration tendency *P*_*E*_ (see below), (ii) personal information about the rewards associated with that resource type (*a*_*ir*_), and (iii) whether the forager has been socially stimulated by seeing another forager eat that resource type *P*_*S*_ (see below). During evaluation of a resource item, these three factors come together to determine the probability *P*_*F*_ to choose to eat that item as follows:

1 $$\begin{array}{*{20}l} P_{F} &= P(r | a_{ir}, a_{ie}, \sigma_{i}, P_{E}, P_{S}) \\&= min\left[ 1.0, \left(\frac{a_{ir}}{a_{ie}} \right)^{\sigma_{i}} + P_{E} + P_{S} \right] \end{array} $$

where *a*_*ir*_ is the reward forager *i* expects from resource type *r* (personal information based on reinforcement learning), *a*_*ie*_ is an assessment of the quality of resources that can be found in the environment (see below), and *σ*_*i*_ scales selectivity, i.e. how likely an individual selects when *a*_*ir*_<*a*_*ie*_. Since associations are initially zero (*a*_*ir*_=0), unknown resource types can only be sampled via *P*_*E*_ or *P*_*S*_. For solitary foragers this means that *P*_*E*_ must be greater than zero. For grouping foragers, *P*_*S*_ could in principle replace *P*_*E*_ as the means to sample unknown resources. Once *a*_*ir*_>0, $\left (\frac {a_{ir}}{a_{ie}} \right)^{\sigma _{i}}$ contributes to the probability of choosing a certain resource type, which is maximal when *a*_*ir*_>*a*_*ie*_ and less than one if *a*_*ir*_<*a*_*ie*_. If *a*_*ir*_>*a*_*ie*_, the forager is certain to choose the resource item, irrespective of *P*_*E*_ and *P*_*S*_. The impact of *P*_*E*_ and *P*_*S*_ is therefore greatest when resource are relatively unfamiliar (*a*_*ir*_<*a*_*ie*_).

Selectivity is adjusted relative to environmental conditions by adjusting *a*_*ie*_ (Fig. 1d, compare dark gray and black). When a forager’s stomach is not full at DIGESTIONTIME, the forager decreases its environmental expectation: *aie*′=(1.0−*ϕ*_*i*_)*a*_*ie*_; otherwise the expectation is increased: *aie*′=(1.0+*ϕ*_*i*_)*a*_*ie*_, where *ϕ*_*i*_ determines the rate with which *a*_*ie*_ is changed. Each time the forager is too selective, it does not fill its stomach and reduces its selectivity, and vice versa. As a result, *a*_*ie*_ is tuned in order to optimise energy intake, within the constraints of the algorithm. Qualitatively, this selection algorithm can give rise to the optimal food choice rule [44] where only resources above a certain perceived quality are eaten and all others are ignored (zero-one rule). Note however that our algorithm works on perceived quality and not actual quality since the foragers are learning about resource quality and are not omnipotent. Moreover, *σ*_*i*_ can evolve, so that while the zero-one rule is possible, it need not evolve.

**Satiation aversion:** foragers develop temporary aversions after becoming satiated (stomach filled) with a given resource type. Satiation aversion causes foragers to completely ignore that resource type for one DIGESTION cycle (100 minutes) after which the aversion disappears. Satiation is common in foragers like primates that consume many secondary ‘toxic’ compounds [45], and/or require a balanced diet [46]. This model specification ensures that foragers consume a diverse set of resource types [31].

### Learning

In the absence of any social influences on learning, learning in our model is composed of (i) exploration, (ii) reinforcement learning about rewards associated with resources, and (iii) skill learning. All foragers start life without any knowledge about resources, and so do not have any expectation about energy rewards (*a*_*ir*_=0) nor any resource processing skill. To enable foragers to sample (partially) unfamiliar resource types, and hence to start learning, we implemented exploration. After processing resource items, foragers develop skill, which increases the rewards they can obtain from resources items of that type. After consuming resource items, foragers develop expectations about rewards via reinforcement, and can use those to decide what to eat.

**Exploration:** The probability that a forager explores an item of resource type *r* is:

2 $$ P_{E} = P(r | \varepsilon_{i}, c_{ir}) = \varepsilon_{i} (1 - c_{ir})  $$

*ε*_*i*_ is the exploration rate, and *c*_*ir*_ is the certainty with which forager *i* assesses the reward of resource type *r*. Certainty was included to ensure that foragers do not continue exploring when already highly familiar with resources. For completely unfamiliar resources *c*_*ir*_=0 and there is no certainty. However, when rewards from resource types no longer change, for instance because skill levels are high, certainty becomes high, and foragers end up with a low tendency to explore that resource type. Certainty *c*_*ir*_ is updated as follows:

3 $$ c_{ir}'=(1-\lambda_{i})c_{ir}+\lambda_{i}\left(1-min\left(1.0,\left|\frac{e_{ir}-a_{ir}}{e_{ir}}\right|\right)\right)  $$

where *e*_*ir*_ is the reward forager *i* obtains from resource *r*, and the same learning rate (*λ*_*i*_) and discrepancy (*e*_*ir*_−*a*_*ir*_) are used as during updating of expected rewards (see Eq. 6).

**Skill learning:** A forager *i*’s skill *s*_*ir*_ for processing a specific resource *r* is a function of experience *t*_*ir*_ and ‘task difficulty’:

4 $$ s_{ir} = \frac{t_{ir}^{S_{r}}}{H_{r}^{S_{r}} + t_{ir}^{S_{r}}}  $$

which is 0 when *t*_*ir*_=0 and tends to 1 when *t*_*ir*_ becomes very large. *t*_*ir*_ is the total time a forager *i* has spent processing a resource type *r* in its life, and increases each time the forager processes and consumes a resource item of type *r*.

Skill *s*_*ir*_ determines the reward *e*_*ir*_ forager *i* obtains from resource type *r* as a function of resource quality *Q*_*r*_:

5 $$ e_{ir} = Q_{r} s_{ir} + N(0, Z)  $$

where *N*(0,*Z*) represents environmental noise, where a value is drawn from a normal distribution with mean 0 and a standard deviation of *Z* (0.005). Resource types with high *H* (Fig. 1c, dashed lines) take longer to learn, while resource types with high *S* have a shallow increment in rewards during initial learning (Fig. 1c, gray lines).

**Reinforcement learning about expected rewards:** The rewards that foragers associate with each resource type *r* are updated via reinforcement as follows:

6 $$ a_{ir}' = a_{ir} + \lambda_{i}(e_{ir} - a_{ir})  $$

where association *a*_*ir*_ is the reward that forager *i* associates with resource type *r*, *e*_*ir*_ is the energy obtained from resource type *r*, and *λ*_*i*_ is the learning rate. This corresponds to a Rescorla-Wagner model [42] where all stimuli have the same salience. Associations are initially non-existent (i.e. zero), and the reward is obtained immediately after consumption of the resource leading to direct reinforcement.

### Social influences on learning

**Local enhancement (LE):** arises spontaneously through grouping behaviour, since individuals are inclined to approach locations in which other members of their group are found, and thereafter to interact with resources in those regions. We therefore do not directly implement local enhancement, but it emerges spontaneously as soon as foragers move in groups [28]. The local enhancement that we consider is coarse grained, and does not direct individuals to particular resources, or to features of those resources.

For the two other social learning mechanisms, during CHECKSAFE a random ‘demonstrator’ is selected from any neighbors in COPYSPACE (see ‘Local decision making’) that are processing and consuming a resource. The impact of the demonstrator depends on the social learning mechanism.

**Stimulus enhancement (SE):** In addition to selecting resources according to their expected reward and the tendency to explore a given resource type asocially, SE increases a forager’s probability to consume resource type *r* by:

7 $$ P_{S} = P(r | \gamma_{i}, d) = d \gamma_{i}  $$

where *γ*_*i*_ indicates the strength of SE, and *d*=1 if forager *i* observed a neighbor consuming resource *r* within the last 30 min. and otherwise *d*=0. Only one resource type *r* is subject to SE at a time. SE does not directly affect expected rewards or skill.

**Observational learning (OL):** occurs during the action OBSERVE at rate *ω*_*i*_ (see ‘Local decision making’) and allows forager *i* to increase its processing skill for a specific resource type, in proportion to the time spent observing, where the change in experience *Δt*_*ir*_ is:

8 $$ \Delta t_{ir} = max[K \frac{o_{ik}}{M}(t_{kr} - t_{ir}),0.0]  $$

where *K* scales the increase, determining how effective skill copying is, and *o*_*ik*_ is the effective time forager *i* observes neighbor *k*: *o*_*ik*_=*min*[*τ*_*i*_,*p*_*k*_], where *τ*_*i*_ is the maximum time forager *i* decides to spend observing its neighbor, and *p*_*k*_ is the time left for neighbor *k* to complete its present action. Greater observation time leads to greater skill acquisition, where maximal observation time is the maximal time it takes to process and consume a resource (*M*). The increase in the skill level is bound to the skill level of the observed individual, and there is no skill gain if the skill level of the observed individual is lower than, or equal to, the forager’s own skill level. A forager does not know in advance whether a ‘demonstrator’ is highly skilled or not. Observation does not provide information about rewards.

### Energy budget, population turn-over and selection

The energy budget is determined by (i) energy gain due rewards from food intake which depends on learning at every DIGESTIONTIME, (ii) a per minute energy metabolism cost (METABOLISM, see Section 1 in Additional file 1), and (iii) an energy costs of 5000 for a reproduction event, which represents a substantial part of total energy. Energy accumulates if energy intake from food exceeds metabolism and reproduction costs.

Foragers die of old age (at 20 years), stochastically determined deaths, or starvation. Births occur as a function of energy reserves each time a forager dies, keeping the population constant at size *N* (100), where probability that forager *i* reproduces is:

9 $$ P_{R} = P(i | N) = \frac{{h_{i}^{W}}}{\sum_{j=1}^{N} {h_{j}^{W}}}  $$

where *h*_*i*_ is an individuals energy level, *N* is the population size, and *W* (=3) scales the strength of the selection function.

The learning and foraging parameters *δ*_*i*_, *ϕ*_*i*_, *σ*_*i*_, *ε*_*i*_, *λ*_*i*_, *γ*_*i*_, *ω*_*i*_, *τ*_*i*_, are specific to forager *i*. Parameter combinations that lead to greater energy levels lead to faster rates of reproduction. An offspring inherits its parent’s parameters, with a chance of mutation (0.05). In case of mutation, a new parameter value is drawn from a normal distribution centered on the parent’s parameter value, and with a standard deviation that is one fifth of the maximum value of the parameter (see Table 1). Thus parameters can vary between individuals and can evolve over time via inheritance to offspring, mutation and natural selection. The mutation rate was selected operationally such that parameters evolve consistently within a reasonable time frame.

Foragers are born in their parent’s group. There is no migration between groups. The population is inviable if the average energy level does not rise above the minimum energy needed to give birth.

### Emergent dynamics

Since we only define local sensing and behavioral actions of foragers, the development of a forager’s repertoire emerges from its interaction with the environment over time. This environment includes the resources and their distribution, which affects the temporal autocorrelations in encounters with resources. The movement of foragers is characterized by inter-patch travel where no resource items are found, and intra-patch search, assessment and consumption of resource items. Within each patch, a forager has access to the resource types that are present in that patch. Over their lifetime, foragers encounter all patch types and all the resource types they contain, many times, thus there is ample opportunity to consume all resource types repeatedly. On reaching a patch, a forager’s experience with those resource types will depend on previous encounters with those resource types, and if it consumed those resources in the previous digestion cycle it could be satiated with respect to those resource types.

The dynamics of foraging are characterized by learning and food choice [28, 31]. Foragers move through the environment and when they encounter resource items, the food choice algorithm determines whether any are consumed (Eq. 1). Foragers start out exploring various unknown resources (via *P*_*E*_ and/or *P*_*S*_), and as they gain experience about rewards, personal information tends to become more dominant in their food choices. Personal experience is updated after consumption events and includes *a*_*ir*_, the assessment of rewards (Eq. 6) and the increment of skill (Eq. 4) which in turn increases the reward obtained (Eq. 5). Due to consumption of many resources, the expectation of the environment *a*_*ie*_ will increase, increasing the fraction of resources for which *a*_*ie*_ is greater than *a*_*ir*_. This increases selectivity towards resources with high *a*_*ir*_, and can lead to reduced food intake (i.e. a forager’s stomach is no longer full at digestion). At this point *a*_*ie*_ decreases again. Thus the forager’s expectation of the environment *a*_*ie*_ tends to equilibrate on a value in relation to values of *a*_*ir*_, such that the intake of resource items is close to the maximum of 20. This ensures that the forager is eating selectively but still eating close to the maximal number of resource items within each digestion cycle (DIGESTONTIME). The ratio of *a*_*ir*_ to *a*_*ie*_ is therefore similar across simulation types, irrespective of how fast *a*_*ir*_ increases due to differences in skill development time.

The combination of (i) food choice biased to resource types with high *a*_*ir*_ (selective foraging), and (ii) learning via updating of *a*_*ir*_ and *t*_*ir*_, generates a positive feedback.

This positive feedback generates a familiarity bias and a development process that is contingent on stochastic initial conditions, leading to idiosyncratic learning histories and somewhat arbitrary variation between foragers in their knowledge of the environment. Therefore, while learning is biased towards high quality resources, due to an intrinsic familiarity bias in the process, learning can get ‘stuck’ on a self-stabilizing repertoire as soon as this repertoire fulfills the intake needs of the forager [28]. This familiarity bias becomes strong in environments with pure patches, and when foragers do not become satiated after eating a lot of a given food type [28, 31]. We therefore focus on patches with several resources and satiation as a default case, which stimulates foragers to develop diverse diets.

The familiarity bias implies that foragers have greater *t*_*ir*_ for some resources than others, and also a more accurate assessment *a*_*ir*_ of rewards *e*_*ir*_. Since *λ*_*i*_ typically evolves to high values (see Section 4 in Additional file 1), *a*_*ir*_ is generally an accurate estimate of *e*_*ir*_. The main cause for differences in familiarity is therefore differences in *t*_*ir*_ and these determine differences in *e*_*ir*_ and *a*_*ir*_. As a result, the impact of social influences on learning therefore concern (i) biases on choosing resource types, which indirectly affect *t*_*ir*_ in the case of LE and SE, and (ii) direct gains in *t*_*ir*_ in the case of OL.

In groups, the actions of neighbors and group-level dynamics can have indirect and direct influences on food choices and learning [28]. Due to the need to stay in a group (imposed in the model), there is a strong ‘consensus’ or ‘conformity’ effect, where the decision of neighbors to stop or not stop in a patch can affect the feeding opportunities of foragers and hence their learning trajectories. Moreover, the direct observation of neighbors and its effects, depends on what neighbors have decided to eat, or depends on copying opportunities [27]. In turn, the effect of a social stimulus will depend on what an observer already knows, and whether it can find the resource type of interest. If a forager would already choose a resource item on its own accord (*a*_*ir*_>*a*_*ie*_) then *P*_*S*_ would not matter and the social influence would be redundant.

Moreover, *P*_*S*_ can increase the rate of food intake and feedback on selectivity via the updating of *a*_*ie*_.

Thus the impact of evolving parameters, in particular those of exploration and social learning, are not predefined in the model and are the object of study. In previous work, which can be considered a baseline for, and pseudo-replicate of this study in terms of the foraging and grouping parameters, the evolutionary attractors have been established [24, 39, 41]. Our results here are consistent with those findings (see Section 4 in Additional file 1). Here we go beyond these existing models and study how foraging and (social) learning parameters co-evolve.

### Simulations and analysis

To analyze our model we distinguish between different classes of parameters (see Section 2 in Additional file 1). Of the 50 parameters, 22 are independent fixed parameters that are either empirical estimates relevant for primates, or are computationally motivated but still empirically reasonable. 17 additional parameters either follow logically from, or are constrained in some way by, independent fixed parameters, and are also empirically reasonable. This group of 39 fixed parameters sets the empirically motivated spatio-temporal scaling context, including life-history, that is relevant for primates and other small-medium mammals, in which the learning mechanisms that we study are embedded.

Within this context, we focus on the key parameters of interest, namely the 4 evolving parameters that define exploration (*ε*_*i*_), stimulus enhancement (*γ*_*i*_) and observational learning (*ω*_*i*_ and *τ*_*i*_), and the 8 fixed parameters that define grouping (social context for local enhancement and other social influences on learning). To do so we ran evolutionary simulations with solitary populations (*S*, where grouping is switched off) in order to establish an asocial baseline, and then ran three kinds of simulations with grouping: (i) *G*_*LE*_, grouping where only local enhancement occurred; (ii) *G*_*SE*_, grouping where stimulus enhancement (*γ*_*i*_), but not observational learning, could evolve freely; (iii) *G*_*OL*_, grouping where observational learning (*ω*_*i*_ and *τ*_*i*_), but not stimulus enhancement, could evolve freely. In all cases, the exploration rate (*ε*_*i*_) could evolve freely. The remaining 4 evolving parameters (*δ*_*i*_, *σ*_*i*_, *ϕ*_*i*_, *λ*_*i*_) ensure that the foraging and reinforcement learning parameters are not arbitrarily defined, but co-evolve with the main parameters of interest.

To study the effect of the environmental context we vary (i) the task difficulty of resources (*H*_*r*_) and (ii) the rate of environmental change (*EC*). As a default we considered patchy environments with multiple resource types in each patch (mixed patches). For additional sensitivity analysis we tested the main qualitative results in environments with (i) patches with a single resource type (pure patches), (ii) randomly distributed resource types (random), and (iii) environmental change where resource types change in quality *Q*_*r*_, but remain known to foragers (comparable to [20, 29]). We do not vary parameters that define life-history characteristics and spatio-temporal scaling as this is beyond the scope of our species of interest.

Our analysis included two main steps. First we ran evolutionary simulations for 1000 years. In simulations with solitary populations evolvable parameters were initialized on randomly selected values. Simulations with grouping parameters were initialized with evolved parameters from solitary simulations, but could continue to evolve. We repeated this process in each kind of environment, and in each case repeated 10 simulations with different random seeds.

We measured the impact of a particular learning mechanism in terms of average energy levels in the population. To determine the evolved values of parameters we analyze parameter values from ancestors (obtained from ancestor traces) at the end of simulations (year 850–950). We used the averages of 10 simulations to represent the ‘evolved parameters’ for a given condition, and compared their consistency across the different environmental settings (see Section 4 and 6 in Additional file 1). In this way we established ‘evolutionary attractors’ for the set of evolving parameters. In our results we focus on exploration and social learning parameters (*ε*_*i*_, *γ*_*i*_, *ω*_*i*_ and *τ*_*i*_). The results of other evolving parameters do not change the interpretation of the results (see Section 4 in Additional file 1).

Second, to determine why parameters evolved to particular values, and to establish the impact of particular learning mechanisms, we conducted additional analysis using two kinds of non-evolutionary simulations without mutations. ‘Parameter sweep’ simulations were used to study the effect of systematically varying the value of a single parameter (local sensitivity analysis), both across and within groups while keeping other parameters fixed on average evolved values that where relevant for a particular social learning mechanisms and ecological condition. ‘Switch’ simulations were used to study the effect of introducing a particular learning mechanism into a population of foragers initialized with another mechanism. This was done by initializing the population with the average evolved values of parameters for the initial mechanism, with values of parameters relevant for the second mechanism set to zero. The parameter values were then changed to those of the average evolved values of the second mechanisms at the time where the switch was desired.

In these shorter simulations (80–140 years) we measure diet repertoire statistics in more detail: (i) total energy intake $= \sum \limits _{r=1}^{r=R} d_{ir} e_{ir}$, where *d*_*ir*_ is the total number of items of resource type *r* that were consumed by forager *i*, and *e*_*ir*_ is the per item reward obtained; (ii) repertoire quality $= \sum \limits _{r=1}^{r=R} p_{ir} Q_{r}$, and (iii) average skill $= \sum \limits _{r=1}^{r=R} p_{ir} s_{ir}$, where *p*_*ir*_ is the proportion of resource *r* in individual *i*’s diet. Section 3 in Additional file 1 provides further detail about different simulations types.

In sum, while the analysis contains a large number of parameters, the vast majority of these provide a realistic simulation context, and the parameter space for the remaining few is fully explored within realistic bounds.

## Results

### Energy levels

**Fig. 2:**
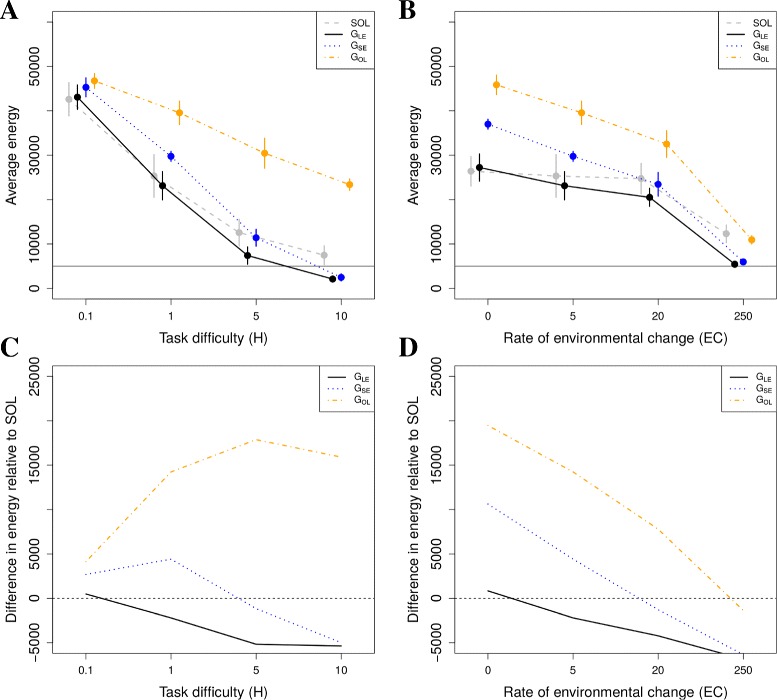
Energy levels and exploration rates. **a** Average evolved energy levels in environments with varying task difficulty (*H*). **b** Average evolved energy levels in environments with varying rates of change (*EC*). **c** Difference in evolved energy levels between solitary (SOL) and other conditions in environments with varying task difficulty (*H*): i.e. all other lines minus the dashed line in top left graph. **d** Difference in evolved energy levels between solitary (SOL) and other conditions in environments with varying rates of change (*EC*): i.e. all other lines minus the dashed line in top right graph. SOL = solitary; *G*_*LE*_ = groups with only local enhancement; *G*_*SE*_ = groups with stimulus enhancement; *G*_*OL*_ = groups with observational learning; Each point (energy level) is the average energy of the whole population between year 950 and 1000 from 10 full evolutionary simulations

We find that the three different social learning mechanisms lead to different evolved energy levels (Fig. 2a and b). Comparison of solitary foragers (SOL) with *G*_*LE*_, which represents grouping effects alone, reveals that grouping either reduces or has no clear effect on energy levels compared to solitary foraging (Fig. 2c and d, solid lines). This happens because grouping leads to greater local competition, as well as coordination problems which reduce foraging efficiency (see Section 5 in Additional file 1). Thus, the coarse-grained local enhancement (LE) that arises in our model does not have a net positive effect on energy levels relative to solitary foraging.

In contrast, both stimulus enhancement (SE) and observational learning (OL) evolve (see Section 6 in Additional file 1 for evolved parameter values), and lead to a large increase in energy levels relative to groups with only local enhancement (Fig. 2a and b, compare dot-dashed and dotted lines to solid lines). These increases can more than compensate for the negative effects of grouping on foraging success (Fig. 2c and d, dotted and dot-dashed lines). The impact of OL exceeds that of SE (Fig. 2c and d, compare dot-dashed to dotted lines), and the combination of SE and OL does not add much relative to OL on its own (results not shown).

These results remain qualitatively the same as we vary foraging task difficulty (*H*, Fig. 2a), and the rate of environmental change (*EC*, Fig. 2b). Both *H* and *EC* increase the difficulty of learning, reducing energy levels (Fig. 2a and b, all lines). *H* increases the time it takes to obtain high rewards from resources, and *EC* reduces the time available to develop skills on resources. As *H* becomes large (Fig. 2c), and as the environment changes more rapidly (Fig. 2d), energy levels in *G*_*SE*_ drop below those of SOL (dotted lines). This implies that the effects of competition and/or coordination problems in groups (see Section 5 in Additional file 1), increase as *H* and *EC* increase. For OL, energy levels drop below SOL when the environment changes too rapidly (Fig. 2d, dot-dashed line). However, as *H* becomes large, OL leads to a large increase in energy intake relative to the other learning mechanisms (Fig. 2c, dot-dashed line). This effect of OL enables group living that would otherwise be inviable (Fig. 2a, dot-dashed line is above horizontal dashed line at *H*=10).

### Exploration rates

**Fig. 3:**
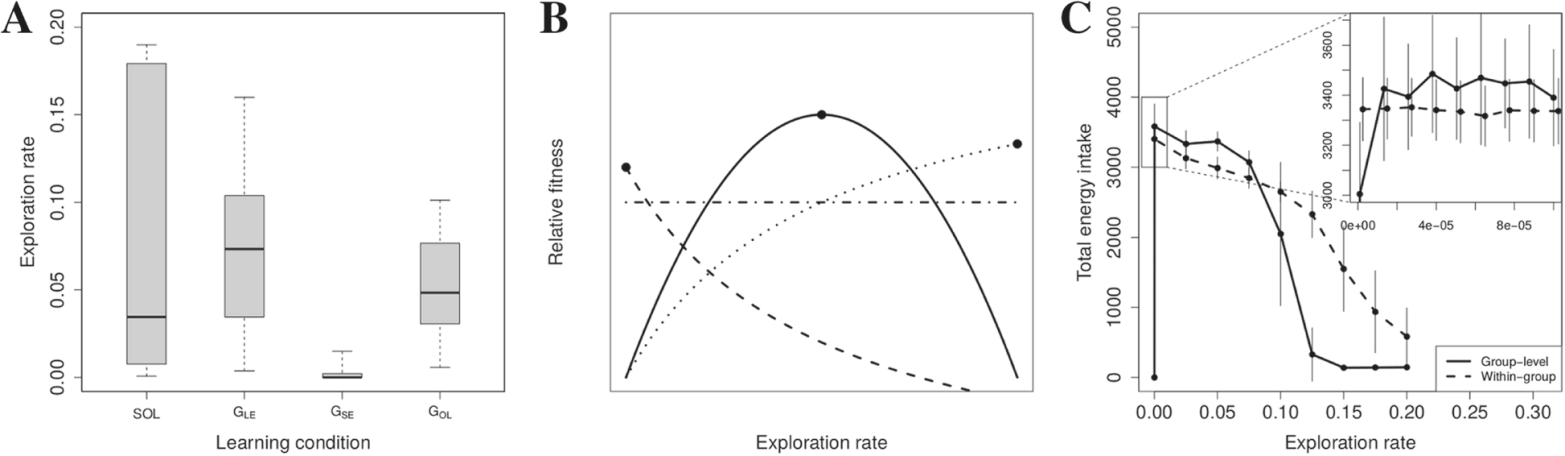
Exploration rates. **a** Evolved exploration rates. SOL: solitary; *G*_*LE*_: groups with only local enhancement; *G*_*SE*_: groups with stimulus enhancement; *G*_*OL*_: groups with observational learning. Data from ancestors between year 850 and 950 from 10 simulations per condition. Box plots show the minimum, 1st quartile, median, 3rd quartile and maximum of means of 10 simulations. **b** Hypothetical scenarios with respect to group-level and within-group optimal exploration rates. *Solid line*: fitness with respect to group-level variation in exploration rates, where the black dot reflects the optimal ‘cooperative’ exploration rate for all foragers in a group; Other lines: fitness with respect to within-group (i.e. individual-level) variation in exploration rates, where the black dot reflects the optimal ‘selfish’ exploration rate for individuals within a group; Dashed line: Information parasitism scenario where the individual-level optimum is below the group-level optimum, and hence individuals have an incentive to explore less than other members of the group; Dotted line: Hypothetical opposite to information parasitism where the within-group optimum is above the group-level optimum and individuals have an incentive to explore more than other group-members; Dot-dashed line: Neutral scenario where there is no real individual-level optimum and no incentive to explore less or more than other group-members. The latter two scenarios are inconsistent with information parasitism. **c** Comparing energy intake between group-level (*solid*) and within-group variation (*dashed*) from simulations. Inset is a zoom in *of main panel*. These results are consistent with the dot-dashed line in B and indicate the absence of information parasitism. Data are means and standard deviation of energy intake obtained using ‘parsweep’ simulations using data from years 40 to 140 from 10 simulations

The three different social learning mechanisms lead to the evolution of different rates of exploration (Fig. 3a). Relative to solitary populations, exploration rates remain more or less the same in groups with LE or OL, but SE leads to very low exploration rates. Below we expand on why SE leads to low exploration rates and how SE and OL lead to increased energy levels.

### Stimulus enhancement

The finding that SE can lead to increased energy levels, without implementation of any special social learning strategy, contradicts the prediction of the aforementioned game-theory that average payoffs in the population do not increase as social learning evolves (Rogers’ paradox), as would be expected if there was information parasitism. However, this raises the question of how the low evolved exploration rates in case of SE are to be explained? To answer this question, we considered two hypotheses.

First, we test the hypothesis that low exploration rates evolve due to information parasitism. Information parasitism can only arise if there is an incentive to explore less than group members because social learning enables the costs of exploration to be avoided. To determine if there is such an incentive, we compared the optimal group-level exploration rate to the optimal individual-level exploration rate. The optimal group-level exploration rate is where the information production (as a public good) is optimized and forms the baseline against which we can compare the success of information parasites.

A hypothetical group-level fitness optimum is shown in Fig. 3b (black dot on solid line), relative to which there are three hypothetical predictions for individual-level fitness at the group-level optimum. First, given information parasitism there should be an incentive to explore less than other group members, fitness should decline as exploration rate increases, and the individual-level fitness optimum should be at a lower exploration rate than the group-level optimum (Fig. 3b, black dot on dashed line). Second, fitness could increase with exploration rate, which would result in the individual-level fitness optima being at a greater exploration rate than the group-level optimum (Fig. 3b, black dot on dotted line). Third, there could be no incentive at all to explore more or less than group members, and so individual-level fitness would be constant (Fig. 3b, horizontal dot-dashed line). The last two cases are inconsistent with information parasitism.

Using ‘parameter-sweep’ simulations (see ‘Simulations and analysis’), we systematically varied the exploration rate across groups to determine the group-level optimum, and within groups to determine the individual-level optimum. Other parameters were set to average evolved levels of simulations with SE. We find that the group-level and individual-level optima are similar when considering a large range of exploration rates (Fig. 3c, main graph, solid and dashed lines; the optima are where energy intake is maximal, i.e. at the very lowest exploration rates). A detailed examination of very low exploration rates (Fig. 3c, inset), revealed that in simulations with group-level variation only, the group-level optimum is found at low but positive exploration rates (solid line). For individual-level variation, exploration rates can be zero because foragers can copy the explorative choices of neighbors (Fig. 3c, inset, dashed line). Critically, however, we do not observe differences in energy intake in this range of exploration rates (Fig. 3c, inset, dashed line is horizontal). Thus, differences between foragers in the number of explorative events are so small that they do not result in different energy intake. Our results are therefore consistent with the scenario where there is no incentive to explore less (or more) than fellow group members and as a consequence no information parasitism (Fig. 3, horizontal dot-dashed line).

Having found no evidence for the information parasitism hypothesis, we turn to the second hypothesis: SE is beneficial because it generates opportunities for new learning outcomes and low exploration rates evolve in order to optimize this effect of SE. This leads to two predictions: (i) the outcome of learning with SE will be beyond what can be achieved in groups with only local enhancement (*G*_*LE*_), and (ii) this alleviation of limitations is maximized at low exploration rates.

To determine whether limitations on learning outcomes are alleviated by SE and how this depends on exploration rates, we focus on repertoire quality and skill levels. Using ‘parameter-sweep’ simulations (see ‘Simulation and analysis’) we determine how skill levels and repertoire quality depend on exploration rates. We do this for groups with only local enhancement (*G*_*LE*_) and groups with SE (*G*_*SE*_), and compare them to determine whether the addition of SE alleviates limitations on learning.

**Fig. 4:**
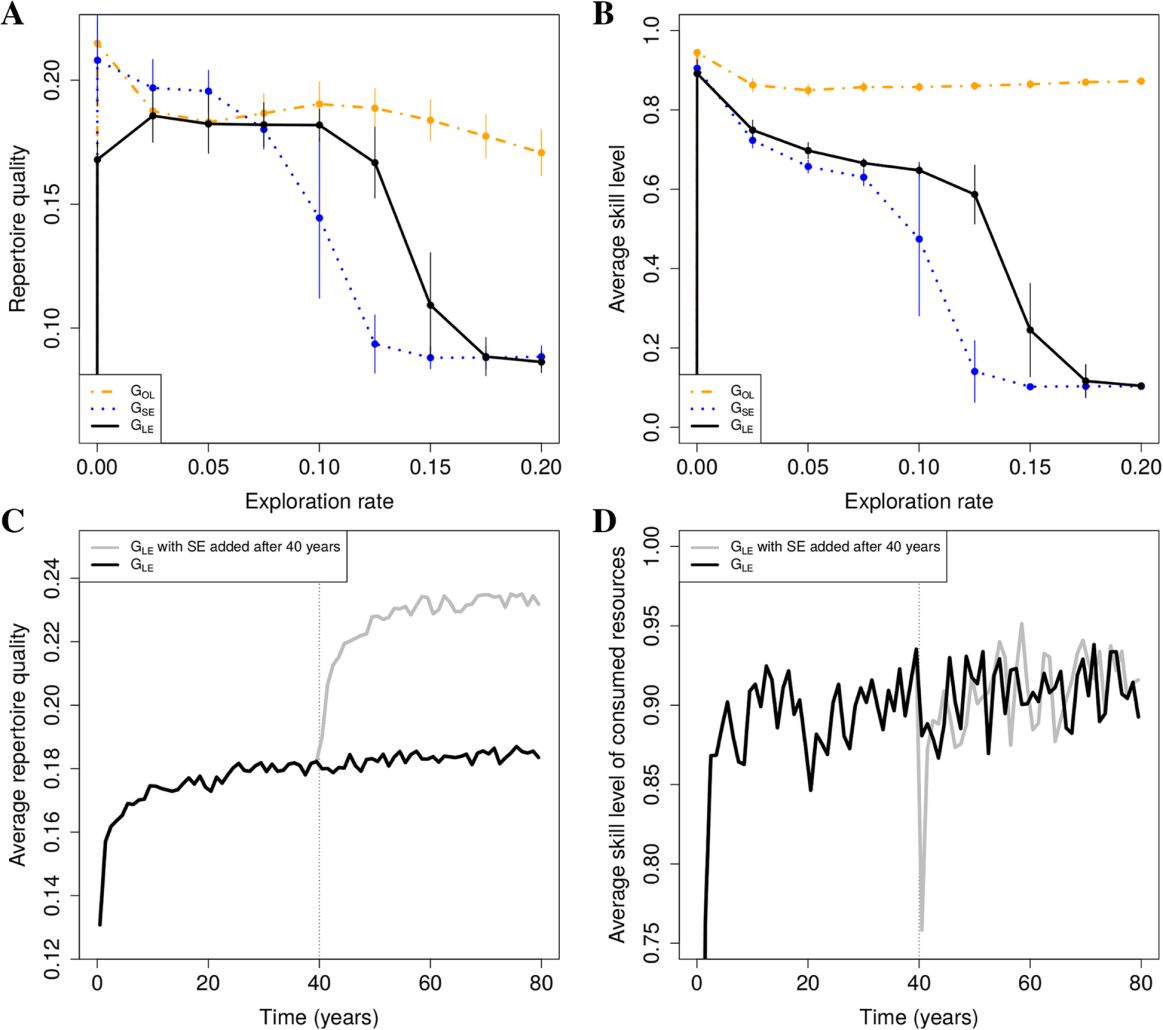
Effect of SE and OL. *Top*: average repertoire quality (**a**) and average skill level of consumed resources (**b**) as a function of exploration rate. Shown are means and standard deviation using data from years 40 to 140 from 10 simulations. Simulations as in Fig. 3C (group-level variation). *Bottom*: ‘switch’ simulations showing the immediate and long term effects of SE on repertoire quality (**c**) and average skill levels (**d**)

For groups with local enhancement we find that high repertoire quality requires sufficiently high exploration rates, otherwise high-quality resource types are not discovered (Fig. 4a, solid line). However, skill levels decline as exploration rates increase (Fig. 4b, solid line), since learning effort becomes spread over many resource types in the environment due to an increase in repertoire diversity. Thus, the comparatively high evolved exploration rates in *G*_*LE*_ (Fig. 3a) are a compromise between limitations on achieving a combination of high repertoire quality and high skill level.

In contrast, for *G*_*SE*_ high repertoire quality at low exploration rates is possible (Fig. 4a, dotted line) and repertoire quality exceeds the highest level attained in *G*_*LE*_ (Fig. 4a, compare dotted to solid line). As a result a combination of high repertoire quality and high skill levels can be achieved, revealing that the limitations that exist in *G*_*LE*_ have been alleviated by the addition of SE. This alleviation of limitations is optimized at low exploration rates, explaining why exploration rates evolve to low values when SE evolves (Fig. 3a).

The alleviation of limitations can be understood by results that were obtained using ‘switch’ simulations (see ‘Simulations and analysis’) where we started with groups with only local enhancement, and after a 40 year burn-in period, subsequently implemented SE. We find that SE leads to an immediate increase in repertoire quality (Fig. 4c, gray line increases immediately after year 40), but an immediate short-term decrease in the average skill of consumed resources (Fig. 4d, gray line decreases immediately after year 40). That SE leads to a bias to consume high-quality resource types can be understood by the fact that neighbors are selective and consume resources of relatively high-quality. Copying opportunities are therefore automatically biased to high-quality resource types (as in [27]). Since different foragers know about different high-quality resource types, by copying each other they select an even higher-quality subset of resource types, explaining how high-quality repertoires can be achieved even when exploration rates are low.

However, because a neighbor’s high-quality choices are unfamiliar to the forager that copies, the average skill level of consumed resources initially drops (Fig. 4d), leading to an initial decrease in average reward (results not shown). Initially, SE is therefore maladaptive, although this may depend on the difficulty of learning. On the longer term, foragers develop skill for those unfamiliar high-quality resource types, and become better able to assess their value. Hence both repertoire quality and average skill levels continue to increase over a period of 20 years (Fig. 4c and d, gray lines increase between year 40 and 60).

These results confirm that low exploration rates evolve in order to optimize the beneficial effects of SE that arise because SE generates opportunities for novel learning outcomes. The introduction of social learning through SE therefore leads to enhanced information production.

### Observational learning

The advantage of OL is the potential for faster skill development. Foragers deploying OL therefore develop greater skill levels, across the full range of exploration rates investigated (Fig. 4b, dot-dashed line). For individuals reliant on LE and SE, high exploration rates typically lead to low skill levels (Fig. 4b, solid and dotted lines), because diets are broadened and development time becomes spread out across a greater number of resources types in the environment. OL directly mitigates this effect by speeding up skill development, with the consequence that increasing values of *ε*_*i*_ have a weaker effect on repertoire quality and average skill compared to LE and SE (Fig. 4a and b, dot-dashed line). Thus, for OL exploration rates do not evolve to low levels (Fig. 3a), and we again do not find evidence for information parasitism.

The relative benefits of OL are not retained across all rates of environmental change (Fig. 2d, compare dot-dashed to dotted and solid lines), which implies that when environments change rapidly, even with OL skill cannot always accumulate. However, if environmental change is slow enough, foragers can accumulate skill by copying each other and inexperienced foragers can catch up with experienced ones through OL. The more difficult that skill development is (i.e. the greater the value of *H*), the greater the potential impact of OL relative to SE and LE, which reflects the value of OL in facilitating skill development for difficult tasks (Fig. 2c, compare dot-dashed to dotted and solid lines). Thus by creating opportunities for rapid skill development, OL allows more efficient resource exploitation, with corresponding increases in repertoire quality and energy intake.

### Generalizations

To examine whether our results generalize to environments with different resource distributions, we also considered environments with randomly distributed resources and environments with pure patches. We again find that (i) SE leads to enhanced energy levels, and (ii) OL increases energy levels even further (see Section 7 in Additional file 1). As before, very low exploration rates evolve when SE, but not OL, is introduced.

We also considered a type of environmental change where resources do not disappear, but only change in quality *Q*_*r*_ (see Section 8 in Additional file 1). This kind of environmental change has a much milder impact, because learned skills are not rendered redundant since resource types no longer disappear. We therefore find that energy levels decline to a much lesser extent than when resource types disappear, and OL can help optimize behavior quite extensively. As before, SE, but not OL, leads to the evolution of lower exploration rates.

## Discussion and conclusions

Overall we find a high degree of mechanism specificity with respect to the impact of the evolution of social learning on rates of exploration, energy returns and skill learning. Our results indicate that (coarse-grained) local enhancement does not directly benefit foraging success, and probably should be regarded as a byproduct of grouping, which likely evolves for reasons unconnected to social learning. In contrast, stimulus enhancement and observational learning can be highly beneficial through enhancing foraging efficiency across a wide range of ecological settings, including those with rapid environmental change (Fig. 2a and b). Moreover, our analyses do not support the assumption that the evolution of social learning leads to information parasitism in general. Rather, for group foragers learning what and how to eat, the evolution of social learning generates opportunities for new learning outcomes.

### Information parasitism and general models

To evaluate the predictions of game-theory models of social learning, we focused on the exploration rate as the parameter determining ‘asocial learning’, since exploration determines the number of resources that are sampled and for which foragers develop reward assessments and skill. For ‘social learning’ we focused on the parameters defining the different mechanisms. If the game theory models correctly capture what is going on in our model, then we predict that there should be an incentive to explore less than group members, and that exploration rates should decline when social learning evolves. This should happen if social learning avoids the costs of exploration. The second prediction is that, because reduced exploration would lead to reduced information production, the average payoffs in the population should not increase as social learning evolves (Rogers’ paradox). Both these predictions are relevant if there are no special social learning strategies, as is the case in our model.

Based on our results, we conclude that the predictions of the game theory models about information parasitism do not generalize to the case we study. Our results show that the evolution of OL and SE actually lead to increased payoffs in the absence of any special social learning strategies, in contradiction of Rogers’ paradox. Moreover, we find that exploration rates do not decline in the case of LE and OL. For SE, exploration rates decline because this enhances skill development. We therefore do not find any evidence for an incentive to explore less than group members, nor that information acquisition via social learning leads to reduced exploration rates, for any of the social learning mechanisms.

To understand why information parasitism does not arise in our model, we re-evaluated how the details of the learning mechanisms related to the main game theoretic assumptions that generate information parasitism. We conclude that the main reason why information parasitism does not arise in our model is because we explicitly implemented a learning process that is gradual. In contrast, in game theory models a lifetime behavior [15], or a given behavioural action [20], becomes fully developed in one time step and information production is very discretized. With gradual learning, information production is not limited to exploration, but is the combined outcome of explorative sampling, reinforcement learning and skill development. In our opinion, there are two main implications that follow from the assumption of gradual learning.

The first implication is that, if learning is gradual, observable foraging behaviour is not necessarily fully developed and therefore social learning does not automatically avoid the costs of exploration. This is because without any special copying strategies, foragers not only copy choices involving fully developed behaviors, but also exploratory choices involving developing and unfamiliar behaviors, and experience the same costs as those group members making exploratory choices. In contrast, in game theory models it is either directly assumed that these costs are avoided (e.g. [15]), or indirectly, when assuming that individuals cannot copy individuals that are in the process of developing or discovering a new behaviour (e.g. [20]). For foragers in our model to avoid copying explorative choices of group members, we would need assume that foragers are capable of distinguishing between neighbors performing fully developed and explorative behaviors, and then bias their copying to only copy fully developed behaviors. Since foragers in our model have no a priori information about what constitutes a fully developed behavior, making this distinction does not appear to be straightforward. In contexts with gradual learning, the assumption that individuals can avoid copying explorative choices therefore implies a relatively complex copying strategy. We therefore argue that the general relevance of the assumption that social learning ‘avoids costs’ of asocial learning is questionable in contexts where learning is a gradual and protracted process.

The second implication of gradual learning is that information production is nearly unavoidable during social learning because there is no clear trade-off between social learning and information production. Instead, social influences are integrated into the learning process, and even non-explorers can produce information. Even if only one forager of the group is exploring (*ε*_*i*_>0), all other foragers that copy the explorer can still produce information for the group through reinforcement learning and skill development. Selective foraging decisions make this information available to other foragers. Thus, even if there was an incentive to explore less than group members, reduced exploration would not necessarily lead to reduced information production.

In sum, the major predictions and assumptions of the game-theoretic models are not supported by our analysis, suggesting that their conclusions may not always be robust. Instead, our results emphasize that the evolution of social learning can be highly mechanism specific: only stimulus enhancement leads to the evolution of low exploration rates, and only local enhancement is not obviously beneficial. This raises an open question about which social learning mechanisms are being represented in modeling approaches that only define social learning at a functional level and where mechanisms are not specified. Our findings therefore contribute to a body of theory which emphasizes the importance of including mechanistic detail in evolutionary analyses [23–26, 47, 48] and supports the idea that models are not necessarily generalizable just because they lack detail [40, 49].

Due to the specificity of our model, we cannot confidently predict model outcomes beyond the context of group foraging in environments with a diverse set of resources. Future multi-scale research will have to determine whether or not in other learning contexts, and/or with other learning mechanisms, the payoffs and information production processes that lead to information parasitism, are generated. By comparing various such models we can begin to identify generalities across contexts and mechanisms, and assess to what extent they correspond with the assumptions and predictions of game-theoretic models. In this way we could move away from a process where generalizations are assumed, to one where they are demonstrated.

We hope that our model and its outcomes will stimulate empirical studies to test whether and under which conditions our findings are valid in real world systems. Following the approach we have taken for the analysis of our model, empirical studies might be able to vary individual exploration rates experimentally to infer related individual and group-levels benefits (Fig. 3b). Independently of the specific approach, important new insights could be gained through experimental investigations of which learning conditions, and for which social learning mechanisms, trade-offs arise between exploration and social information use.

### The (co-)evolution of social learning mechanisms

As LE arises due to grouping, and (at least, as implemented here) SE and OL can only exist in groups, researchers seemingly need to take the evolution of grouping into account when considering the evolution of social learning. Our results can be therefore be interpreted with respect to two evolutionary scenarios, in which grouping evolves because of the effects of social learning, or grouping evolves for other reasons, such as alleviating predation risk [50], as is typically thought to be the case in primates [51, 52].

In the context we study, grouping would probably not evolve for the benefits of LE in the context of learning what and how to eat in groups, since LE does not enhance foraging success (Fig. 2a and b). In most cases, LE due to grouping is either neutral (environments with pure patches, no environmental change and/or very low task difficulty), or maladaptive, compared to solitary foraging. As coarse-grained LE is nearly inevitable [28] and LE in general appears to be widespread in nature [2], we conclude that LE is probably not an adaptation, but rather is a byproduct of grouping that evolves for some other reason. This conclusion potentially resolves the ‘conundrum’ surrounding LE [53]: how can we understand the prevalence of LE when it is seemingly not adaptive? However, we cannot rule out the possibility that LE could be adaptive in other contexts, such as food patch detection in small groups [39].

In the present skill learning context, grouping could, however, evolve for the benefits brought about through SE and OL, as energy levels in groups with SE and OL can exceed those in solitary foragers (Fig. 2a and b). In that case, SE and OL, and enhanced capabilities thereof, would need to co-evolve with grouping, but would not be expected to evolve where resource types are replaced too rapidly. However, where grouping evolves for reasons other than social learning, we would expect SE and OL to evolve under a wide range of conditions, including rapid environmental change, and where resource processing is sufficiently difficult (i.e. large *H*).

SE and OL are beneficial because they generate opportunities for novel learning outcomes. That SE is beneficial in the context of skill learning is striking, given that SE does not directly involve any copying of skill. The benefits of SE arise because stimulus enhancement enables groups to develop high-quality repertoires (Fig. 4a, compared dotted to solid line) by generating an automatic bias to copy the high-quality choices of neighbors, enabling exploration rates to evolve to low values. The low exploration rates favors skill development (Fig. 4b), leading to a combination of high-quality repertoires and high skill levels that is not possible in groups without copying. Similarly, observational learning leads to the accumulation of skill in groups, which substantially exceeds the capacities of single foragers. OL tends to have a far greater impact on energy levels than SE, suggesting than SE may become redundant once OL can evolve (Fig. 2a and b). It is possible that observational learning in animals arises through a combination of the mechanisms that we implement as SE and OL.

The cognitive prerequisites of SE and OL may be an important determinant in their evolution. While not implemented in our model, we anticipate that OL might exert greater demands on cognitive processing than SE. For instance, at the extreme, OL could represent production imitation, perhaps even requiring theory-of-mind or perspective taking. Conversely, SE is not likely to be cognitively demanding, and may well be explained by domain-general attention processes [54]. Consistent with findings of Arbilly and Laland [53], we thereby expect SE to be widespread in nature.

To the extent that the evolution of OL requires the evolution of specialized cognitive capabilities, those capabilities are candidates for social learning adaptations. Two different scenarios are plausible here. First, the cognitive prerequisites of OL may be domain-specific and evolve primarily to implement the specific social learning mechanism. In this case, the costs of these cognitive prerequisites (not considered here) will directly affect the evolution of the mechanism. Second, the cognitive prerequisites of OL may be domain general and may evolve for other reasons. In this scenario, the evolution of a specific social learning mechanism becomes tied to the evolution of those other aspects of behavior (e.g. social cognition, physical cognition, other learning processes) that are affected by cognitive abilities. In the latter case, a comprehensive understanding would require researchers to study the evolution of OL together with other relevant factors that could plausibly lead to selection on the associated cognitive capabilities, and hence the co-evolution of multiple traits.

Developmental mechanisms are also an important consideration in the evolution of SE and OL. We implement SE and OL as tendencies that are fixed over a lifetime. However, animals could plausibly ‘learn to socially learn’ [55], including through domain-general mechanisms [54]. If such learning would be governed by immediate rewards, however, our analysis points to difficulties in how it would arise. To the extent that our model is realistic, it implies that the immediate direct effect of SE is a reduction in average rewards since foragers tend to become biased to resources for which they have low skill (Fig. 4d), while the direct effect of OL is a time cost and no reward. In principle, foragers could therefore learn to associate the choices of others with low rewards, in which case learning to socially learn might be more challenging than it first appears, at least in a ‘skill learning’ context. Where this occurs, we would predict SE and OL to be underpinned by attentional or motivation biases, which, together with the aforementioned cognitive pre-requisites of OL, may be regarded as social learning adaptations. The question of whether the evolution of social learning requires the evolution of social-learning-specific adaptations remains one the major unresolved issues in the field, but it is likely that the answer will depend on the social learning mechanism involved.

Finally, because they can generate opportunities for novel learning outcomes, we draw attention to the capacity of SE and OL to play an important role in opening up novel ecological niches that are not accessible for solitary foragers, or to groups without SE or OL. This is clear from the way that OL enables groups to become viable at very high task learning difficulty (*H*=10, Fig. 2a). Since the threshold at which resources become too difficult to exploit through asocial processes alone is somewhat arbitrary, a similar argument can be made for SE, but with a more limited scope. Particularly OL, but also SE, enable skill levels in groups to accumulate beyond the level obtainable in groups without copying, even in changing environments. Where this skill accumulation occurs beyond the level that solitary foragers can achieve (as in *H*=10), then it can potentially open up novel niches. In this kind of scenario, group foragers could exploit niches with increasingly difficult resource processing. If such niche expansion occurs as a function of accumulated skill, then viability becomes dependent on group knowledge, i.e. cultural niche construction [56].

## Additional files

Additional file 1 Supporting information. This file includes additional detail about the main simulation model and the modeling methodology and additional analysis. (347 KB PDF)

Additional file 2 Video 1. Video showing group foragers with only local enhancement. Each forager is indicated by a SEARCH area (gray semi-circle), REACH (gray circle) and a movement trajectory (red to blue line). For illustration purposes, the resource items are shown as colored circles, and patches by a larger gray circles. Each patch can be assumed to be a distinct patch type, with unique resource types (different colours within a patch). (7390 KB MP4)

Additional file 3 Video 2. Video showing group foragers observing each other, which relevant for both stimulus enhancement and observational learning. Each forager is indicated by a SEARCH area (gray semi-circle), REACH (gray circle) and a movement trajectory (red to blue line). When a forager observes another forager the foragers are connected by an olive-green line. For illustration purposes, the resource items are shown as colored circles, and patches by a larger gray circles. Each patch can be assumed to be a distinct patch type, with unique resource types (different colours within a patch). (2370 KB MP4)

## Abbreviations

LE: Local enhancement
OL: Observational learning
SE: Stimulus enhancement
