# Supplementary material for: Skill learning and the evolution of social learning mechanisms
Source: BMC Evol Biol. 2016 Aug 24;16(1):166. doi: 10.1186/s12862-016-0742-9 (PMC4995764; doi:10.1186/s12862-016-0742-9)
Supplement: Additional file 1 — Supporting information. This file includes additional detail about the main simulation model and the modeling methodology and additional analysis. (347 KB PDF) [file 12862_2016_742_MOESM1_ESM.pdf]

Supporting information for “Skill learning and the Evolution of Social Learning Mechanisms”

Daniel J van der Post[1*], Mathias Franz[2], Kevin Laland[1]
1 Center for Social Learning and Cognitive Evolution, St Andrews University, St Andrews, UK
2 Leibniz Institute for Zoo and Wildlife Research, Berlin, Germany
∗ E-mail: d.j.vanderpost@gmail.com

Contents

1 Detailed model description 2
1.1 Purpose . . . 2
1.2 State variables and scales . . . 2
1.3 Process overview and scheduling . . . 3
1.4 Design concepts . . . 5
1.5 Specification choices of scaling context . . . 5
1.6 Initialization . . . 6
1.7 Input . . . 6
1.8 Submodels . . . 6
1.8.1 Forager continuous updates and decision-making algorithm . . . 7
1.8.2 Behavior actions of foragers . . . 7
1.8.3 Forager discrete updates . . . 9
1.8.4 Resources . . . 10
1.9 Comparison to diet model by van der Post & Hogeweg . . . 10

2 Classes and inter-dependencies of parameters 11
2.1 Independent fixed parameters . . . 11
2.2 Dependent and partially dependent parameters . . . 12
2.3 Evolving parameters . . . 13
2.4 System control parameters . . . 13

3 Further details on simulations 15

4 Evolution of learning rate and foraging parameters 16

5 Effect of grouping on foraging efficiency 19

6 Social learning parameters 20

7 Random and pure patchy environments 22

8 Alternative environmental change 24

1 Detailed model description

Our modeling choices are based on the following observations of primate-like foragers: (i) there is a diverse environment with different potential food or resource types of varying quality (Smuts et al., 1987; Chapman and Fedigan, 1990; Altmann, 1998); (ii) resources are limited in their abundance and are depleted when consumed, or contain aversive secondary compounds, such that foragers need to consume a variety of resources (Provenza, 1996); (iii) foragers live in groups with overlapping generations (Smuts et al., 1987); (iv) foragers need to learn about the rewards that are associated with particular resources (Matsuzawa and Hasegawa, 1982, 1983; Birch, 1999; Visalberghi et al., 2003; Hardus et al., 2014); (v) foragers are selective (i.e. do not eat everything they encounter) (Chapman and Fedigan, 1990; Altmann, 1998); (vi) foragers can develop skills associated with processing particular resources and so obtain greater rewards from those resources (Byrne, 1999; Jaeggi et al., 2010); (vii) foragers can observe the foraging choices of their neighbors and this can affect the foragers' subsequent choices (Visalberghi and Addessi, 2003; Fragaszy and Visalberghi, 2004; Agostini and Visalberghi, 2005; Jaeggi et al., 2010; van de Waal and Whiten, 2012; van de Waal et al., 2014). These empirical observations directly determine the main features of our model, and we study the dynamic processes that they generate. Here we document a full model description based on the ODD protocol for describing simulation models (Grimm et al., 2006).

1.1 Purpose

We develop an event-based, individual-based, spatially extended model of group foragers learning what and how to eat, in order to enable a multi-scale approach to the evolution of social learning mechanisms. Specifically, we study how the evolution of individual-level social learning parameters affects learning outcomes through self-organizing processes. We address the role of self-organization in the evolution of social learning, its mechanism specificity and scrutinize the assumptions made in game-theoretic models.

1.2 State variables and scales

Our model consists of a 2-dimensional environment with resource items and foragers. Space and time are continuous (via event-based scheduling), but some variables are updated via discrete time steps where each time point is a minute, a day is 720 minutes (only daylight), and a year is 360 days.

Resource types are described by: a spatial position, a maximum energy value Q_r, a task difficulty H_r and S_r which determines how rapidly foragers can initially learn to process a resource type. Specific resource items are also defined by a spatial position and whether they have been consumed this year or not. To generate resource patches, resource items are arranged randomly in a circle around a randomly selected patch center. Resource patches are characterized by: a location, a radius, resource density. The "patch-level" is emergent in the sense that patches can only be observed by foragers by sensing resource items. For random environments, items are placed singly at any random location.

Foragers are characterized by the following state variables: age, energy, position, heading, behavior state, behavioral action, time to complete an action (completion time), memory (see

below), having a particular feeding target (i.e. a food item), a group identity, and parameters that determine behavior (see Table S1). Forager memory includes: (i) having a particular feeding target (i.e. a resource item), (ii) associations a_{ir} between a resource and a reward, (iii) experience t_{ir}, the total time spend processing a particular resource type, (iv) a particular resource type for which the forager has a stimulus for via stimulus enhancement, (v) an estimate of the quality of resources that can be found in the environment a_{ie}, and (vi) estimates of how certain foragers are about resources c_{ir}. The forager population is fixed, but the number of groups changes when groups die and are 'born'.

1.3 Process overview and scheduling

We distinguish two levels of scheduling: (i) continuous time and (ii) discrete time steps (every minute).

Discrete updating: every minute we update individual age, reduce energy due to energy metabolism, determine if an individual dies due to a fixed death rate, starvation or old age, and if so select a parent according to energy levels:

$$({h_i}^W/(\sum_{j=1}^{N} h_j))^W \tag{S1}$$

where h_i is an individuals energy level, N is the population size and $W(=3)$ is the selection coefficient. Groups die when the last group member dies, and are born when a group reaches the maximum group size (20 foragers) and is randomly split into two equally sized daughter groups. Mutations in behavior parameters during reproduction generates genetic variation. If variation affects reproductive success, natural selection arises. Each resource type is checked if it is replaced by a new resource type every minute according to EC the rate of environmental change. Every DIG_TIME (100) minutes all consumed resource items are 'digested'. If any resource type exceeds SATIATION_FRAC*MAX_STOMACH number of items, then the forager attains a temporary satiation aversion for that resource type for SATIATION_TIME minutes.

Continuous updating: every behavioral action has a continuous time point at which it is completed (completion time). Actions can therefore end anywhere within a minute (with the resolution of "float" types in C++ programming language), and an action started in a previous minute is completed in the next minute. All foragers are put into a queue according to their completion time. The individual with the lowest completion time is next to complete its action and choose a new action. The completion time represents the duration of an action. Foragers therefore start their actions asynchronously, but action durations can overlap. This is an event-based scheduling. The sequence of actions of a forager is determined by the decision-making algorithm.

When foragers consume resource items those items are tagged as 'consumed' and become available again the following year. When assessing resources items locally, any consumed resources items are checked whether they have become available again. If so they are tagged as available. This set-up was chosen for computational reasons, such that updating resource items occurs in a 'need to know' manner in relation to what foragers are observing locally. As a result we do not need to update all resource items all the time.

Name	Description	Values
DAY	minutes of daylight foraging time	720
YEAR	number of days	360
PATCH_RADIUS	Radius of the patch	10 m
PATCH_RES	Number of resource types in a given patch type	5
PATCH_RES_SEL	Subset of possible resource types in particular patch	3
R	Number of resources species	250
N	Population size	100
W	Selection coefficient which determines the strength of selection	3
MAX_AGE	Maximum age that foragers can reach	20 years
DEATH_RATE	Rate of stochastic death	0.1 per year
BIRTH_ENERGY	Energy invested in offspring	5000 units
DIGESTIONTIME	Time interval at which digestion occurs	100 minutes
STOMACH_CAP	Number of items that can be eaten before forager is full	20
REACH	Distance at which forager can reach a resource item	0.9 m
M	The maximum event time (for EAT, OBSERVE, MOVE)	1 min
COPY_SPACE	Distance at which foragers can observe what their neighbors are doing	20
K	The proportion of the difference in experience that can be learning in skill learning by observation	0.1
SEARCH_ANGLE	Angle about forward heading that the forager searches	$\pi/2$
SEARCH_DISTANCE	Maximum distance at which resource items can be detected	2 m
NUM_ITEMS_PER_SEARCH	Number of items that can be assessed in SEARCH_TIME	20
MOVE_SPEED	Speed when moving forward	3.6 km / h
STEP_DISTANCE	Distance of a single move forward event	1 m
NOTHING_TIME	Duration of NOTHING action	M
EAT_TIME	Duration of EAT action	M
A	Area of the environment	39.2 km^2
DENSITY	Total resource density	0.75
PATCH_ITEMS	Number of resource items in patch	1200
PATCHES	Number of resource patches	24500
Q_r	Maximum energy reward of resource type r	$N(0.1, 0.1)$
H_r	Practice time needed before obtaining half the maximal reward	0.1..10
S_r	Scalar for the sigmoid function describing how rewards increase with practice	1..4
Z	Noise on the reward foragers obtain from a resource: standard deviation of a normal distribution about e_{ir}	0.005
EC_1	Rate at which resource species are replaced by new unknown species	0..R species per year
EC_2	Rate at which resource species change their quality Q_r	0..R species per year
METABOLISM	Energy used for metabolism	0.01 per min
SATIATION_TIME	Time that satiation lasts	DIGESTIONTIME
SATIATION_FRAC	Proportion of stomach filled by a resource type in order to become satiated	0.9
G	Number of foragers in a group	20
SAFE_NEIGHS	Number of neighbors a forager needs to feel safe	9
SAFE_SPACE	SAFE_NEIGHS should be within this distance	17 m
PROB_CHECK_SAFE	Probability to check if safe	0.9
ALIGN_PROB	Probability to align to neighs direction after MOVETOGROUP because not safe	0.9
ALIGN_ZONE	Zone of alignment	20 m
MOVETOGROUP_DISTANCE	Distance individual moves to become safe	3 m
MOVETOGROUP_SPEED	Speed at which forager move to become safe	180 m/min
SEARCH_TIME	Duration of SEARCH action	$0.5 * M$
δ_i	Distance foragers move forward after not finding preferred food	0..20 m
σ_i	Selectivity scalar	0..20
ϕ_i	Rate scalar for updating the expected reward for the environment a_{ie}	0..1
λ_i	Rate scalar for reinforcement learning	0..1
ε_i	Maximum exploration rate	0..1
γ_i	Increment in probability to select a resource if previously stimulated in stimulus enhancement	0..1
ω_i	Probability to OBSERVE neighbor	0..1
τ_i	Maximum time observing neighbor	0.01..1 min
e_{ir}	Reward obtained by forager i from resource r	0..$max(Q_r)$
h_i	Forager's total energy (or health)	0..∞
t_{ir}	Total time forager i has practiced processing resource r	0..MAX_AGE
a_{ir}	Reward forager i expects from resource r	0..$max(Q_r)$
a_{ie}	Reward forager i expects from environment	0..$max(Q_r)$
c_{ir}	Certainty estimate of expected reward	0..1
n_{ik}	How interesting neighbor k is for forager i to be selected as demonstrator	0..1
p_i	The time forager i has left to complete performing its present behavioral action	0..M
o_{ik}	Effective observation time by forager i of neighbor k equals $min(\tau_i, p_k)$	0..M

Table S1: List of parameters and variables. Upper case letters and names: to refer to invariant parameters that do not change during simulations. Lower case letters: variables of foragers that change during their lifetime. Greek letters: parameters that can mutate, but are invariant during a forager's lifetime. Subscripts: i = forager identity; r = resource type; k = forager's neighbor; e = environment.

1.4 Design concepts

Emergence: our model is all about detecting and understanding emerging processes and the interactions between them. The following is a list of emerging phenomena:

- Foraging patterns: larger-scale detection of resource patterns beyond the local perception of individuals (van der Post and Semmann, 2011a,b).
- Patterns of resource depletion (van der Post and Hogeweg, 2006).
- Interactions between grouping and resource patterns (van der Post and Hogeweg, 2006).
- Learning patterns (van der Post and Hogeweg, 2006).
- Cultural phenomena (van der Post and Hogeweg, 2008; van der Post and Hogeweg, 2009).

Adaptation: there are at least two implemented levels of adaptation: (i) individuals have a decision-making algorithm that determines how they respond to the local environment, and (ii) evolutionary adaptation of behavioral parameters via natural selection.

Fitness: we define that the rate of reproduction of foragers depends on energy intake, therefore more feeding generates a greater birth rate. A forager's lifespan depends on a fixed death rate, starvation and maximum age. We study how fitness is affected by the evolution of foraging and social learning.

Prediction: individuals do not predict future conditions, but respond to environmental conditions they encounter. These responses are embedded, in the sense that they are determined by locally observable conditions. We refer to this type of responsiveness as TODO, as in individuals DO WHAT THERE IS TO DO (Hogeweg and Hesper, 1985). What individuals do is determined by evolvable decision-making algorithms.

Sensing: central for TODO is local information processing, leading to larger scale TODO-based pattern recognition beyond the direct perception of the individual (Hogeweg and Hesper, 1991; van der Post and Semmann, 2011a). We only define local perception of food (2 meters), and on a somewhat larger scale the awareness of neighbors.

Interaction: foragers can approach, eat and deplete resources. Foragers can detect each other and approach, align or move away from other individuals.

Collectives: resources patches and groups of foragers.

Observation: we collect data on what foragers eat, what their associations are, their experience, their group members, their age, energy, location, number of offspring, and evolvable parameters. Analysis generally proceeds via macro-observables (e.g. energy level) and becomes increasingly detailed as more information is needed to understand what and how processes are occurring (see main text).

1.5 Specification choices of scaling context

The scaling context of our model is determined by fixed parameters (Table S1):

Spatial and temporal resolution: At the minimal level we define the minimal time and spatial scale. Evolvable action durations can be minimally 10 seconds (t_{MIN}), to keep the model

running fast enough. Space is continuous: individuals and resources can be placed at any given location.

Local information processing: Constraints on movement and perception (local information processing) are defined in terms of the spatial and temporal scale. Maximum speed is defined in terms of the minimal time interval, so that the duration of moving 1 meter can never be below t_{MIN}. The maximum range of resource detection (r_D) is then chosen to be small, so that individuals have to move to detect food (Video 1, gray semi-circle). Within this range all resource items are detected. The individual's REACH d_r is then chosen to be below maximal resource detection range (Video 1, gray small circle). This is quite a reasonable assumption (equal detection range and REACH is probably a limit case).

For grouping we assume that groups are cohesive and assume that forager's can always keep track of all their group-mates, but ignore foragers of other groups. Moreover, for the alignment zone (ALIGN_ZONE) we assume a shorter range because alignment requires identifying the direction neighbors are moving.

Energy and life history: We fix the population at 100, and when a forager dies a parent is chosen according to its energy level E_i. The parent gives birth with cost E_B. This generates a viability threshold for the population, i.e. energy intake must be sufficient to have a net positive energy balance *and* must include sufficient energy for reproduction. If not, average energy levels drop and average energy levels in the population drop below E_B, in which case the population is not viable. In our simulations however, births and deaths continue.

Environment: The environmental settings were chosen to support selective foraging. This means that the resource density is so high that foragers do not end up eating everything, and need to be selective to reach the highest energy levels.

1.6 Initialization

We initialize the environment with a fixed number of resource types and multiple items of each type, but different resource distributions. An initial population of 100 foragers is initialized with a either randomly initialized evolvable parameters for solitary populations, or parameters that have evolved in solitary populations for simulations with grouping. For simulations with grouping the population is initialized as solitary, and after a 40 years start-up period any offspring are grouping phenotypes and groups start to form. Individuals are initialized at random positions, start without an action, and with no information about the environment. Their first action will be FOODSCAN.

1.7 Input

The model does not include any external model or data files of driving environmental variables.

1.8 Submodels

Here we present a "mathematical" skeleton of the model updating schedule:

1.8.1 Forager continuous updates and decision-making algorithm

During each minute:
while any action ends this minute, take soonest ending action:
1) Complete Action
——* MOVE: get new position
——* FOODSCAN: get new food target, or not
——* MOVETOFOOD: get new position
——* MOVETOGROUP: get new position
——* EAT: gain energy $E_i = E_i + e_{ir}$ and gain experience: t_{ir} and a_{ir}
where E_i is individual energy and e_{ir} is energy per food item
——* OBSERVE: gain experience t_{ir}
——* NOTHING: ready for next action
2) Choose New Action based on previous action:
—- if was MOVE: reselect MOVE if not yet reached δ_i meters
—- if NO ACTION YET ASSIGNED
——* CHECK IF SAFE and update any stimulus enhancement
——* if (not safe) then MOVETOGROUP
——* else (safe)
———** if was MOVETOGROUP then align to the direction of group members in ALIGN_ZONE
———** if was SEARCH or MOVETOFOOD then
————-*** if resource target still exists
—————-**** if (distance to target < d_R)
——————-***** EAT
—————-**** else
——————-***** MOVETOFOOD
————*** give up (food is gone)
———** if no action assigned and (RAND < ω_i) then OBSERVE
———** else (no action and not observe)
————*** if (stomach is full)
—————-**** do NOTHING
————*** else
—————-**** if (previous action != FOODSCAN) and not crowded
——————***** FOODSCAN
—————-**** else
——————***** MOVE
3) Get new completion time
4) Place forager in action cue sorted by completion time.

RAND is a random number between 0 and 1, ω_i is the probability to OBSERVE, and d_R is the individual's reach.

1.8.2 Behavior actions of foragers

MOVE:

- Get new position and make sure it is on the grid:
  - $\vec{p'} = d_M\vec{v} + \vec{p}$
    where d_M is the MOVE distance, $\vec{v}$ = normalized random vector, and $\vec{p}$ is the position of the forager.
  - while $\bar{(}p'_x\ or\ p'_y < 0$ or $p'_x\ or\ p'_y >= \sqrt{A})$
    - select new $\vec{v}$ (normalized random vector)
    - $\vec{p'} = d_M\vec{v} + \vec{p}$

    where A is the area of the environment.
- Duration: $d_M * t_M$
  where t_M is the time it takes the forager to move 1 meter.

MOVETOGROUP:

- $\vec{v} = \frac{\sum_{j\neq i}^{n_A} \vec{p_j}}{n_A}$
  n_A is the number of group members and $\vec{p_j}$ is the position of neighbor j.
- $\vec{p'} = d_M\vec{v} + \vec{p}$
  where d_M is the MOVE distance and $\vec{p}$ is the position of the forager.
- Duration: $d_M * t_M$
  where t_M is the time it takes the forager to move 1 meter, d_M is the step-distance.

FOODSCAN (Video 1, gray semi-circle): try to get new food target

- for all n food items (distance $< d_F$ and angle $< a_F/2$ from heading)
  where d_F is the radius of search, and a_F is the search angle about the foragers forward heading.
  - assess a maximum of 20 items
  - for each item decide whether to eat of not based on Equation 1.
- Duration: t_F

MOVETOFOOD: get new position closer to food item

- $\vec{v} = \frac{\vec{f}-\vec{p}}{|\vec{f}-\vec{p}|}$
- d = distance to food where $\vec{f}$ is the position of food item and $\vec{p}$ the position of the forager
- if $d > d_r$
  $\vec{p'} = d_M\vec{v} + \vec{p}$
  else
  $\vec{p'} = d\vec{v} + \vec{p}$

- Duration: distance moved * t_M

EAT: consume food item

- Duration: t_E

OBSERVE: observe neighbor

- Duration: τ_i
- leads to acquisition of experience based on Equation 8.

NOTHING: do nothing

- Duration: t_N

1.8.3 Forager discrete updates

Each minute for all foragers:

- Age: age + 1 minute
- Energy: $E_i = E_i - E_m$
  where E_m is energy metabolism per minute
- Stimulus enhancement time (after stimulus):
  - if (stimulus duration < 30 minutes): stimulus time = stimulus time + 1 minute
  - if (stimulus duration >= 30) no more stimulus
- Reproduction:
  - if (population < 100): select parent according to energy (see Equation S1)
    * Energy change due to birth: $E_i = E_i - E_B$, where E_B is the birth cost.
    * Offspring energy: E_B
    * Inheritance: offspring inherits all evolvable parameters (see Table S1).
    * Mutation: with probability 0.05 each evolvable parameter is mutated. A new value is drawn from a normal distribution about the parent's parameter value within an preset range (see Table S1).
- Death (forager dies if):
  - $E_i \leq 0$
  - age >= maximum age
  - RAND < death rate (equivalent to 0.1 per year)

1.8.4 Resources

For all depleted resource items every year:

- all depleted resource items re-appear (or re-grow).
- resource items that are eaten disappear immediately.

This is not computed by looping over all items all the time, but only when those items are assessed by foragers locally.

1.9 Comparison to diet model by van der Post & Hogeweg

The major changes as compared to the model of van der Post and Hogeweg (2006) are as follows: (i) We now focus on skill learning rather than diet learning with delayed feedback from digestion. To add skill learning, we model rewards to increase over time (Equations 4 and 5 in main text), while in van der Post and Hogeweg (2006) rewards were fixed. Moreover, to simplify the model we excluded delayed feedback, and instead assume that foragers can immediately sense rewards. (ii) Since we include skill learning we can now include observational learning. Previously in van der Post and Hogeweg (2006); van der Post and Hogeweg (2008); van der Post et al. (2009) we had only included local and stimulus enhancement; (iii) We now include evolution of parameters, while in (van der Post and Hogeweg, 2006) parameters were fixed on pre-selected values. To allow for evolution we now focus on larger environments with populations with multiple groups with variable size. In van der Post and Hogeweg (2006) only single groups of fixed size were considered. Since we now include evolution, and aimed to explicitly consider the evolution of exploration rate relative to social learning parameters, we implement an intrinsic exploration tendency that depends on the forager's certainty estimate. In van der Post and Hogeweg (2006) this was implicit by assuming that individuals only explore resources for which they have no existing preference or association with a probability depending on 'trail rate'. The latter could effectively limit exploration via a familiarity-selectivity feedback. This could generate unfair constraints on asocial learning, relative to which an advantage of social learning would be an artefact. Hence we added a more general exploration tendency, mitigated by knowledge of a resource ('certainty', see Equation 2).

Finally, we would also note that the model has been completely recoded in C++, while van der Post and Hogeweg (2006) was originally coded in C.

2 Classes and inter-dependencies of parameters

The parameter space of the model can be evaluated in an informed way by distinguishing between different types of parameter. In particular, inter-dependencies between parameters imply that parameter values are not arbitrary but that the values follow given some logic about how parameters are inter-related (i.e. they must fulfill conditions relative to each other). Thus we distinguish between (i) primary fixed parameters, (ii) dependent fixed parameters, (iii) evolving parameters, (iv) system control parameters. All those indicated with * are either systematically varied, or evaluated in sensitivity analyses.

2.1 Independent fixed parameters

This concerns 22 independent fixed parameters that are either empirically and/or computationally motivated. 10 independent fixed parameters are mainly empirically motivated based on small to medium sized monkeys:

1. DAY, MINUTE, YEAR: standard time, focusing on daylight (primate activity time).
2. MAX_AGE & DEATH_RATE: average (10 years) and maximum age (20 years), which is reasonable for monkey species.
3. DIGESTION_TIME (100 minutes): on the order of hours.
4. REACH (0.9 m): reasonable limitations of reach for primates.
5. MOVE_SPEED: 3.8 km/h reasonable for relaxed primate foraging.
6. PATCH_RADIUS* (10 m): defining the crown of trees in tropical rain forest. Note that very similar results are obtained with smaller patches (van der Post and Hogeweg, 2006)
7. PATCH_RES* (5): number of resource species per tree species (e.g. fruit, seeds, flowers, young and mature leaves).

12 primary fixed parameters are mainly computationally motivated but are also empirically reasonable:

1. POPULATION_ SIZE (100): in order to have fast enough simulations.
2. RESOURCE_DENSITY (0.75 items / m sq): high enough such that there is not too much depletion causing all foragers to learn everything (i.e. not selectivity).
3. number_of_resource_species (250): this is great enough to ensure learning is sufficiently difficult to be interesting as determined in previous work (van der Post and Hogeweg, 2006). It is empirically motivated but underestimates natural diversity.
4. METABOLISM (0.01): set arbitrarily as reference point on which to scale other energy parameters.
5. G* (maximal group size, 20): this is constrained by POPULATION_SIZE, and empirically motivated.

6. STOMACH_CAPACITY (20): this is empirically motivated, but the relatively low number of items is computationally convenient.
7. SEARCH_ANGLE ($\pi/2$): this is empirically motivated (main vision is forward), close to evolved values (van der Post and Semmann, 2011a), and more computationally convenient than 360 degree vision.
8. SEARCH_DISTANCE (2 m): distant-dependent search is empirically motivated, but the discrete cut-off and distance are computationally convenient.
9. NOTHING_TIME (1 min): some maximal time span after which to check a forager's motivation again. Checking only once every minute is computationally convenient.
10. EAT_TIME (1 min): eating for a minute per item is computationally convenient and short enough that stomachs become filled, and empirically reasonable.
11. SEARCH_TIME (30 sec): searching for half a minute is computationally convenient and short enough that stomachs become filled, and empirically reasonable.
12. STEP_DISTANCE (1 m): a meter is computationally convenient minimal distance and does not overshoot SEARCH_DISTANCE, and is empirically reasonable

2.2 Dependent and partially dependent parameters

17 dependent fixed parameters either (i) follow logically based on, or (ii) are constrained in some way by, primary fixed parameters:

1. A (area, 39 km sq): this is constrained given a target density of foragers and POPULATION_SIZE
2. PATCHES* (24500): set so that in principle patches would cover the area considered (i.e. a closed forest canopy), depending on A and PATCH_RADIUS.
3. PATCH_ITEMS* (1200): this follows from PATCHES, and the number of resource items following from A and RESOURCE_DENSITY
4. PATCH_RES_SEL* (3): this follows from PATCH_RES in order to generate some variation across patches (empirically motivated)
5. Q_r (max reward, $N(0.1, 0.1)$): scaled relative to METABOLISM such that net energy gain from food intake is possible. Note that this is further scaled by H_r and S_r over time.
6. Z (noise on rewards, 0.005): scaled relative to Q_r.
7. BIRTH_ENERGY (5000): scaled relative to net energy gain from food items to define the costs of birth, and so the time needed to recover energy levels, chosen such that foragers cannot immediately reproduce again.
8. SATIATION_TIME (100 min): scaled relative to DIGESTION_TIME since satiation is defined in terms of digestion cycles.

9. SATIATION_FRAC (0.9): just a small deviation from complete stomach contents.

10. SAFE_NEIGHS*, SAFE_SPACE*, PROB_CHECK_SAFE*, ALIGN_PROB*, ALIGN_ZONE*, MOVETOGROUP_DISTANCE*, MOVETOGROUP_SPEED*: these parameters are all set relative to each other to define cohesive grouping based on previous work (van der Post and Hogeweg, 2006; van der Post and Semmann, 2011a; van der Post et al., 2015).

11. NUM_ITEMS_PER_SEARCH (20): scaled relative to SEARCH_TIME and with a preference for a low number for computational efficiency. Very low numbers make search very inefficient, and very high numbers are redundant.

2.3 Evolving parameters

8 evolving parameters are not fixed, but simulated evolution explores this parameter space:

1. δ_i (move distance, see 'Local decision making')

2. σ_i (selectivity during food choice, Equation 1)

3. ϕ_i (update factor of expected rewards in the environment, see 'Food choice algorithm')

4. λ_i (learning rate, Equation 3 and 6)

5. ε_i (exploration rate, Equation 2)

6. γ_i (stimulus enhancement, Equation 7)

7. ω_i (rate of observing neighbors, see 'Local decision making')

8. τ_i (duration of observing neighbors, Equation 8).

2.4 System control parameters

14 system control parameters are varied in a systematic way to vary the social, learning mechanism and environmental settings and to conduct sensitivity analysis:

1. Task difficulty: by varying H_r between 1 and 10. At 1 learning is nearly instantaneous, while at 10 solitary foragers are not viable (too difficult).

2. Environmental change: by varying $EC1$ or $EC2$ between 0 and 250. At 0 these is no change, and at 250 solitary forager are not really viable.

3. Resource distributions: we compare our default setting to random and pure patchy environments. Thus we vary a 'meta-parameter' composed of PATCHES, PATCH_SIZE, PATCH_RES and PATCH_ITEMS. Previous work has shown that this targets the key feature of interest, namely local variation in resources (van der Post et al., 2009).

4. Solitary and grouping: here we change the 'meta-parameter' of sociality involving G, SAFE_NEIGHS, SAFE_SPACE, PROB_CHECK_SAFE, ALIGN_PROB, ALIGN_ZONE, MOVETOGROUP_DISTANCE, MOVETOGROUP_SPEED.

In addition, we exclude particular social learning mechanisms by setting the respective parameters of those mechanism to zero and by not letting them evolve.

3 Further details on simulations

In our analysis we use different kinds of simulations:

(i) Evolutionary simulations. These form the backbone of the analysis, and provide the evolved parameters that we use to compare the evolved mechanisms in non-evolutionary follow-up simulations (see below). We first established an asocial learning baseline by evolving foraging and learning parameters in solitary populations. In this way we established the level of optimization achieved via asocial learning and the optimized asocial rate of exploration. Simulations with solitary foragers (SOL) were initialized with randomized values for all evolving parameters. Next we used the average evolved parameters from solitary populations to initialize simulations where foragers form groups. In the GR NC condition no extra parameters evolve. In the GR SE and GR OL condition the relevant extra parameters evolve and are initialized at zero. We also included a GR OL SE condition, but results are qualitatively similar to the GR OL condition, so do not present the results in the main paper.

We ran evolutionary simulations for 1000 years (roughly 100 generations) and repeated 10 simulations of a given setting (with different random seeds). As a default, we did this in environments with a population of 100 foragers and a resource density of 0.75 items per meter squared, which means 24500 patches with 1200 resource items each. This resource density was chosen such that global depletion of resources was limited in order to make sure that foragers would have to specialize in order to optimize their behavioral repertoires. These environmental conditions were also used for other simulations. In evolutionary simulations we varied H (0.1,1,5,10) and EC (0, 5, 20, 250). For non-evolutionary simulations (see below) we focused on $H = 1$ and $EC = 5$.

(ii) Parameter-sweep simulations. Here we used the evolved parameters for a given learning mechanisms, but varied exploration rate in order to (a) determine whether there was evidence for information parasitism, or (b) characterize constraints on learning. These simulations were run for 140 years (40 year burn-in period) which was found to be sufficient for the measure of interest, where the mutation rate was set to zero. In simulations with groups, exploration rate was both varied across groups where we had a population of 9 groups of 11 foragers, or within groups, with a population of 11 groups of 9 foragers. We therefore varied exploration rate over 9 intervals between a minimum and maximum value. In simulations with solitary foragers, ε_i was simply varied across the population (121 intervals).

(iii) Switch simulations. Here we used evolved parameters for a given learning mechanism, and ran simulations for a 40 years burn-in period, after which we changed learning mechanism. In particular we started with groups without copying (GR NC) to generate a relevant pattern of experience in groups, and then introduced stimulus enhancement in order to determine the immediate and more long term effects of SE.

4 Evolution of learning rate and foraging parameters

We observe that learning rate λ_i evolves to a range of about 0.5-1.0 (Fig. S1A and S2A). These are fairly high learning rates, and emphasizes that foragers learn such that a_{ir} quickly match e_{ir}, leading to quick selectivity. Foragers also react a lot to noise, both overestimating and underestimating resources quickly.

We observe that move distance δ_i evolves to around 15 in SOL, and about 10 in groups (GR)

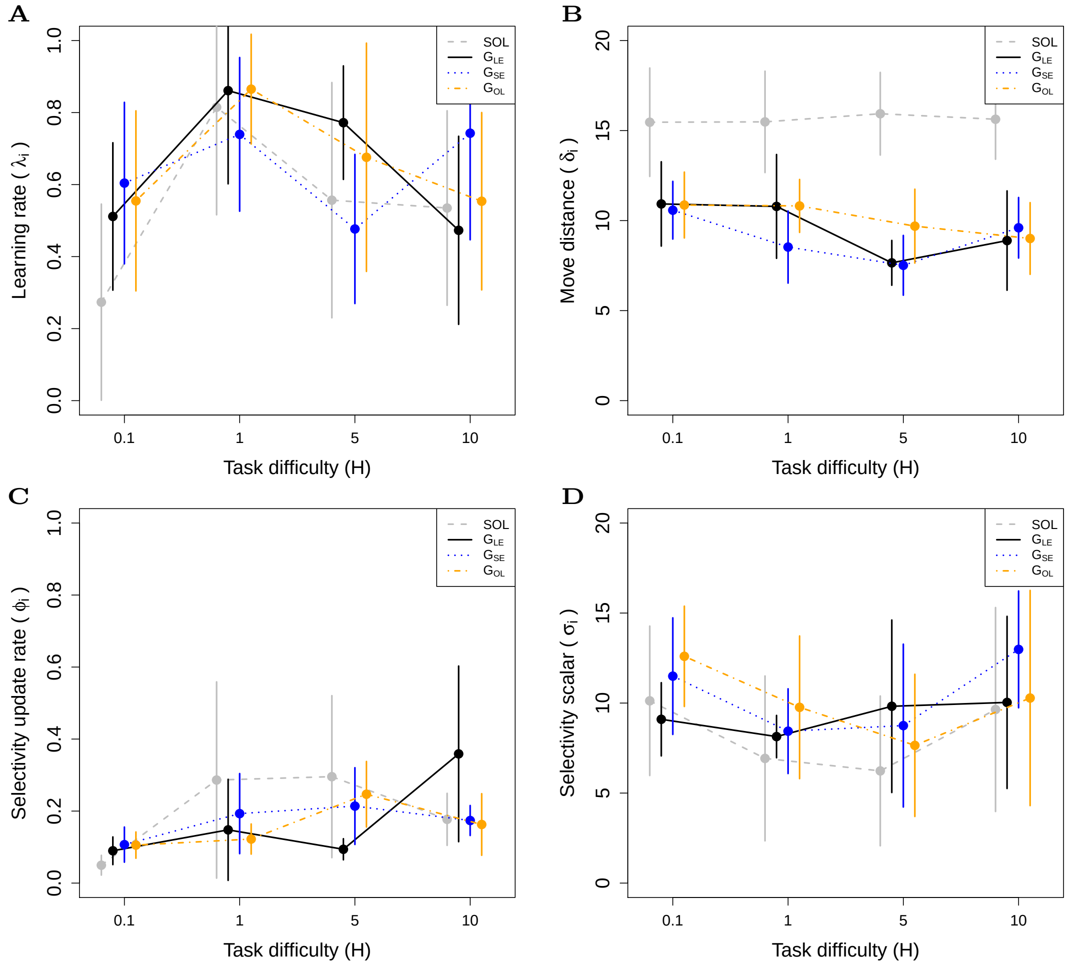

Figure S1: **Evolution of parameters in environments with varying H.** Top left: learning rates λ_i; Top right: move forward distance δ_i; Bottom left: update rate ϕ_i of a_{ie}; Bottom right: selectivity scalar σ_i. SOL = solitary; GR groups; LE = only stimulus enhancement; SE = stimulus enhancement; OL = observational learning. Default environment (orange): 250 resources, mixed patches, 0.75 resource items per meter squared, 100 foragers, max group size = 20, 5 resource types change per year, $H = 1$. Data: pooled data of last 50 years of the whole population (energy of individuals or diets recorded each half year). Whiskers show standard deviation.

(Fig. S1B and S2B). Moving forager a long distance optimizes the time allocation between movement and search in patchy environments (van der Post and Semmann, 2011a). Moving forward a long distance can exacerbate coordination problems in groups, hence the decrease of δ_i in groups. This decrease can limit foraging selectivity, since moving forward a long distance before searching reduces the number of potential sampling events (van der Post and Hogeweg, 2008).

We observe that the update rate ϕ_i of a_{ie} evolves to a range of about 0.05-0.4 (Fig. S1C and

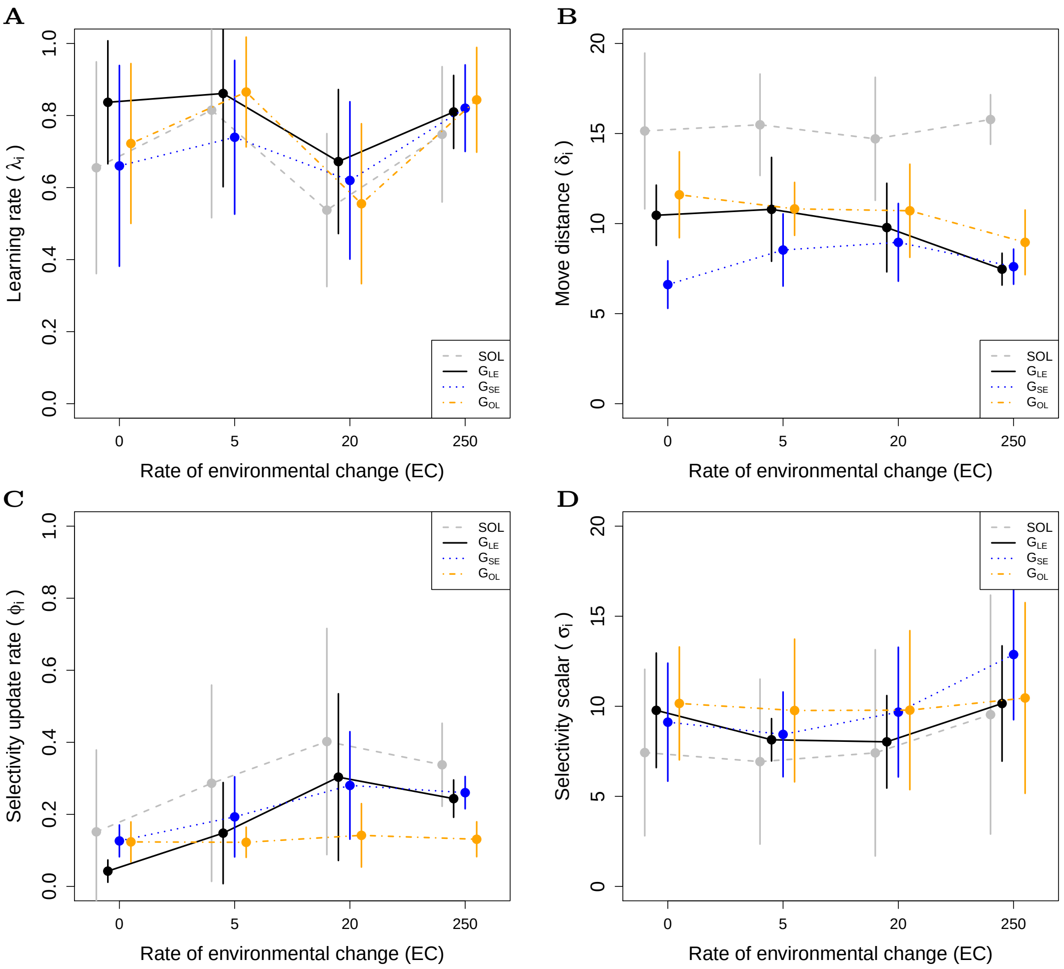

Figure S2: **Evolution of parameters in environments with varying EC.** Top left: learning rates λ_i; Top right: move forward distance δ_i; Bottom left: update rate ϕ_i of a_{ie}; Bottom right: selectivity scalar σ_i. SOL = solitary; GR groups; LE = only stimulus enhancement; SE = stimulus enhancement; OL = observational learning. Default environment (orange): 250 resources, mixed patches, 0.75 resource items per meter squared, 100 foragers, max group size = 20, 5 resource types change per year, $H = 1$. Data: pooled data of last 50 years of the whole population (energy of individuals or diets recorded each half year). Whiskers show standard deviation.

S2C). This parameter determines how quickly foragers alter their selectivity when either having eaten too little or too much. That this parameter evolves to relatively low values (below 0.5), indicates that adjustment of selectivity occurs relatively subtly.

We observe that selectivity scalar ω_i evolves to a range of about 5-15 (Fig. S1D and S2D). These values ensure selectivity, but also some sampling and exploration of less preferred resources (this is reduced when values reach 20). Note that these values are in the range of those shown to generate diet traditions and cumulative cultural diet optimization (van der Post and Hogeweg, 2008).

5 Effect of grouping on foraging efficiency

We tested the effect of grouping on energy intake by comparing solitary foragers (SOL) to foragers living in groups with only local enhancement (G_{LE}). To rule out the effect of grouping on learning, we considered foragers with complete knowledge so that only the possible effects of (i) local foraging competition and (ii) inefficient travel between patches, remain as effects. In Fig. S3 we show the results, where we plot the box plots of the means of 10 simulations of SOL and G_{LE} simulations. The means are calculated over all individuals between year 40 and 140. We find that SOL have greater energy intake than G_{LE} (Wilcox test, W = 81, p-value = 0.01854). This confirms that grouping reduces foraging efficiency independent of the effects of learning. This does not rule out that changes in foraging due to grouping feedback on learning reducing efficiency even further.

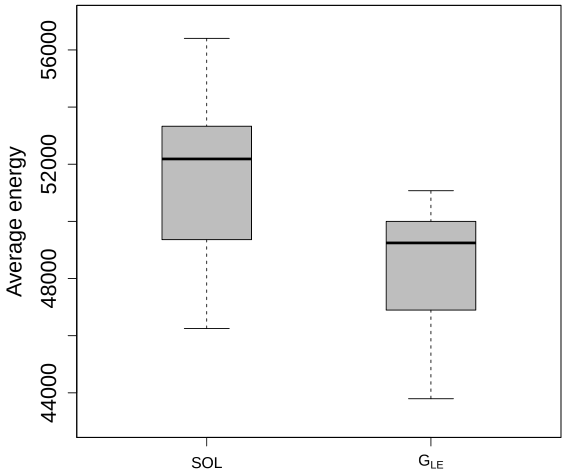

Figure S3: Average energy levels in 'complete knowledge' populations of solitary (SOL) and grouping foraging with only local enhancement (G_{LE}). Foragers were initialized with complete knowledge, which means that foragers have perfect expectations about resource rewards ($a_{ir} = e_{ir}$) and are maximally experienced (t_{ir} =maximal age). We used groups of size 10. The means are calculated over all individuals between year 40 and 140.

6 Social learning parameters

SE evolves via γ_i, and in general evolves to values between 0.2 and 0.6 (Fig. S4A and D). SE effectively overrules personal preferences, i.e. overrules selectivity, thus relying on the selectivity of another forager. Very high values of SE can therefore cause pathological social contagion, where any action by any group member compulsively spreads throughout the group. Since SE helps groups foragers to specialize on resources eaten by their neighbors, and reduces knowledge diversity, increased SE makes sense in a more difficult (high H) environments, although this effect only seems to occur for SE on its own (blue line, top left), but not when in combination with OL (red line).

OL evolves via ω_i and τ_i, which both affect the total time foragers invest in observing their neighbors (middle and right). This time investment tends to increase as environments becomes more difficult (Fig. S4B and C) and change more rapidly (Fig. S4E and F). In those cases the

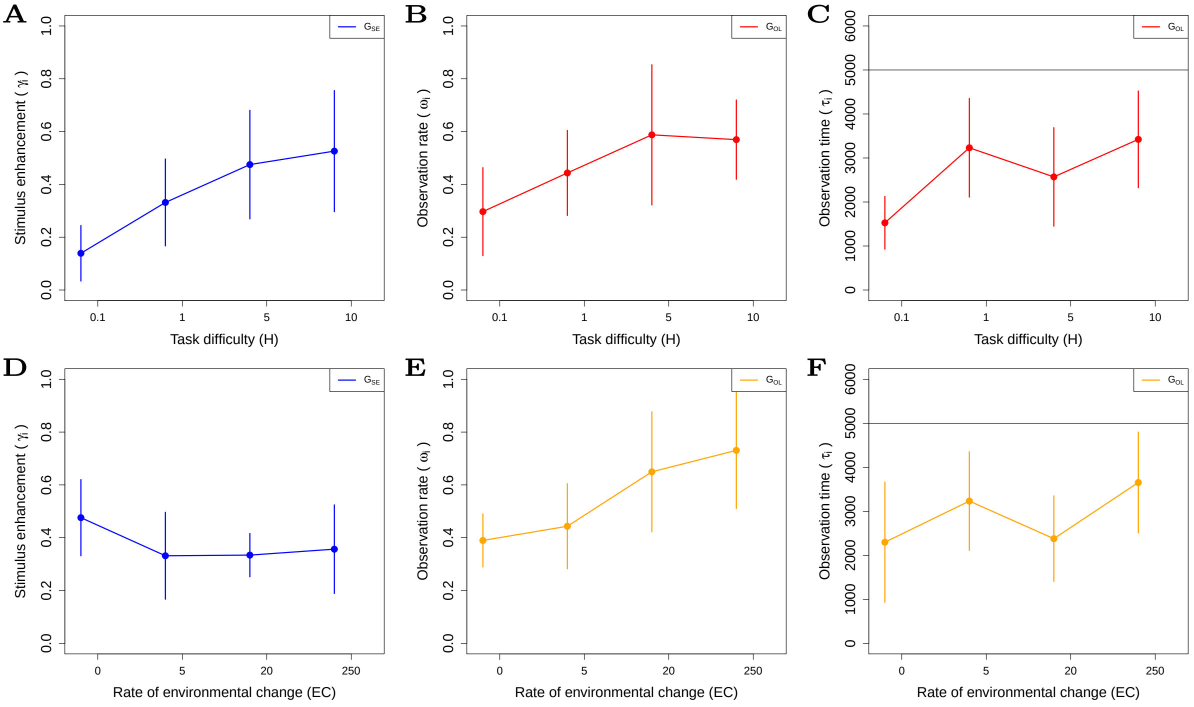

Figure S4: **Evolution of SL parameters in different environments:** average values of γ_i (left), ω_i (middle) and τ_i (right). Top: Environments of varying difficulty. Bottom: Environments with varying rates of change. SOL = solitary; GR groups; LE = only local enhancement; SE = stimulus enhancement; OL = observational learning. Default environment (orange): 250 resources, mixed patches, 0.75 resource items per meter squared, 100 foragers, max group size = 20, 5 resource types change per year, $H1$. Data: pooled data of last 50 years of the whole population (energy of individuals or diets recorded each half year). Whiskers show standard deviation.

benefits of more rapid specialization are high, less easily leading to redundant OL and therewith outweighing the time costs involved in skill development.

7 Random and pure patchy environments

To compare different resource distributions, we compare our default mixed patches setting with two extremes, namely random and pure patches. For random environments, each item is place at any randomly selected location in continuous space. For pure patches, each patch type has a radius of 10 meters and only consists of a single resource type. Patches of each patch type are placed at a random location in continuous space, and can overlap. In all cases we make sure that resource density is 0.75 items per meter squared, so that the amount of food is the same in the different resource distributions.

In random and mixed patchy environments, but not for pure patches, we find that G_{LE} exhibits lower energy levels than SOL (Fig. S5A, compare dashed gray to solid black line). In environments with local variation in resources, random environments and mixed patches, each resource type is more rare, and this intensifies local competition for high quality resources. However, in environments with pure patches, where this is no local variation in resources, which both reduces local competition as well as coordination issues. As in mixed patches, we find that the evolution of SE leads to increased energy levels in random and pure patches environments

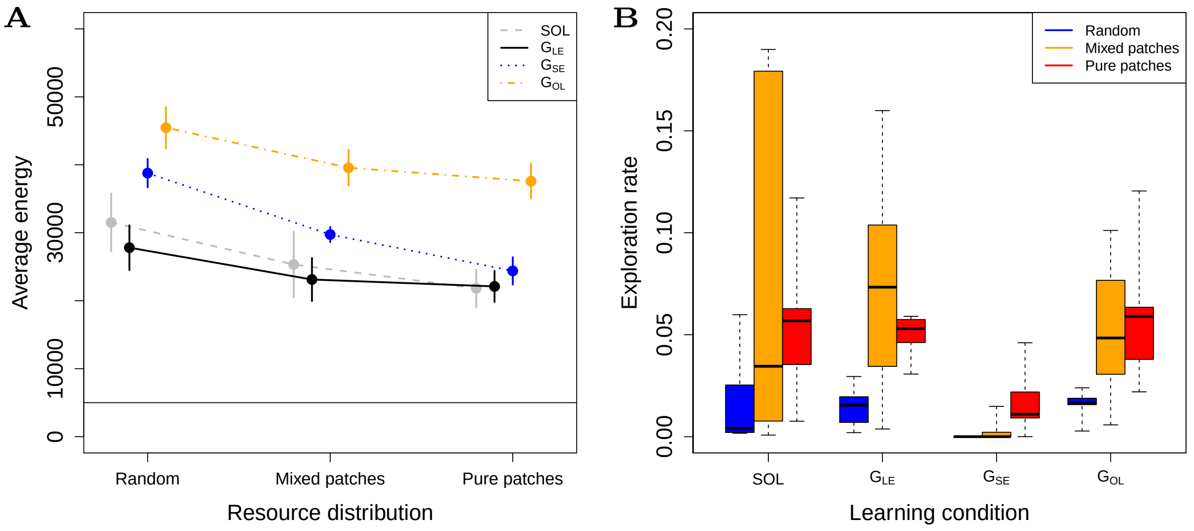

Figure S5: **Evolved energy levels (A) and exploration rates (B) environments with different resource distributions.** A: average energy levels foragers in last 50 years of simulation in environments with random (blue), mixed patches (orange), or pure patches (red). Default environment (orange): 250 resources, mixed patches, 0.75 resource items per meter squared, 100 foragers, max group size = 20, 5 resource types change per year, $H1$. Data: pooled data of last 50 years of the whole population (energy of individuals or diets recorded each half year). Whiskers show standard deviation. B: box plots showing evolved exploration rates in ancestors between year 850 and 950 in environments with random (blue), mixed patches (orange), or pure patches (red). SOL = solitary; Groups; G_{LE} = no individual-level social learning; G_{SE} = stimulus enhancement; G_{OL} = social skill learning. Box plots show medians, upper and lower quartiles, and whiskers show the complete range.

(Fig. S5A, compare blue to black and gray lines). In addition, OL leads to an even greater increase than SE (Fig. S5A, compare orange to other lines).

For exploration rates, we find that same overall pattern that for each environmental condition exploration rates only decline when SE evolves (Fig. S5B). We also find that the exploration rate declines in random environment (Fig. S5B, compare blue to orange and red). This probably relates to exploration opportunities, where resource types are more locally abundant at any one place and time due to high diversity in random environments. Hence, for the same exploration parameter, the effective exploration rate (i.e. eating something that is unfamiliar) will be greater in the random environment than in patchy environments. Thus for the same optimal effective exploration rate, the optimized exploration parameter would be somewhat lower in random environments.

8 Alternative environmental change

In Fig. S6 we show the effect of a different kind of environmental change ($EC2$), namely where resource do not disappear but only change in quality Q_r, where a new quality is drawn randomly from a normal distribution about 0.1 with standard deviation 0.1 (i.e. the same distribution from which resources are initialized). We compare our main environmental change ($EC1$, resources disappear and are replaced by novel ones) at rates $EC5$ (blue line) and $EC250$ (orange line), to the alternative type $EC2$ at rate $EC250$ (red lines). While $EC1\_250$ leads to an overall decline in energy levels relative to $EC1\_5$, $E2\_250$ has a much more reduced impact and even leads to an increase in energy levels when OL is added to the model. Moreover, while the impact of SE is relatively minor for $E2\_250$, it still helps to optimize behavior somewhat relative to LE despite the rapid environmental change.

The reason that OL can enhance energy levels in $EC2$ relative to much slower rates of change for $EC1$ is because with $EC2$, individuals do not loose their processing skills. Hence experience can still accumulate in groups, and foragers can simply track changes in resource quality. When a resource changes in quality, all foragers adjust their assessment of it, and switch to other resources. Discovering resources that recently increased in reward, may be a bit more difficult,

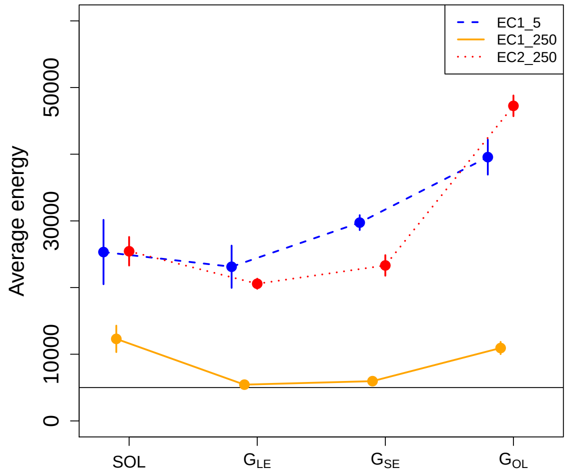

Figure S6: **The effect of alternative environmental change.** We compare changes where resources disappear $EC1$ (blue and orange lines), and where only quality changes $EC2$ (red lines). Average energy in the population; Right: Average values of ε_i in ancestor traces. SOL = solitary; Groups: G_{LE} = only local enhancement; G_{SE} = stimulus enhancement; G_{OL} = social skill learning. Default environment (orange): 250 resources, mixed patches, 0.75 resource items per meter squared, 100 foragers, max group size = 20, 5 resource types change per year, $H1$. Data: pooled data of last 50 years of the whole population (energy of individuals or diets recorded each half year). Whiskers show standard deviation.

but would happen as long as there is a small chance that foragers explore less familiar resources. The latter is the case, given that exploration rates remain high (Fig. 3A). Thus, overall skill can continue to accumulate until foragers would obtain maximal rewards from resources, and all they would have to do is shift according to changes in quality.

References

Agostini, I. and E. Visalberghi, 2005. Social influences on the acquisition of sex-typical foraging patterns by juveniles in a group of wild tufted capuchin monkeys (Cebus nigritus). *Am J Primatol* **65**:335–51.

Altmann, S. A., 1998. Foraging for survival. The University of Chicago Press, Chicago.

Birch, L. L., 1999. Development of food preferences. *Ann. Rev. Nutr.* **19**:41–62.

Byrne, R., 1999. Cognition in great ape ecology: skill-learning ability opens up foraging opportunities. In H. O. Box and K. R. Gibson, editors, *Mammalian social learning: Comparative and ecological perspectives*, pages 333–350, Cambridge, UK. Cambridge University Press.

Chapman, C. A. and L. M. Fedigan, 1990. Dietary differences between neighboring *Cebus capucinus* groups: local traditions, food availability or responses to food profitability? *Folia Primatol* **54**:177–86.

Fragaszy, D. and E. Visalberghi, 2004. Socially biased learning in monkeys. *Learn Behav* **32**:24–35.

Grimm, V., U. Berger, F. Bastiansen, S. Eliassen, V. Ginot, J. Giske, J. Goss-Custard, T. Grand, S. Heinz, G. Huse, et al., 2006. A standard protocol for describing individual-based and agent-based models. *Ecological modelling* **198**:115–126.

Hardus, M. E., A. R. Lameira, S. A. Wich, H. de Vries, R. Wahyudi, R. W. Shumaker, and S. B. J. Menken, 2014. Effect of repeated exposures and sociality on novel food acceptance and consumption by orangutans. *Primates* .

Hogeweg, P. and B. Hesper, 1985. Socioinformatic processes: MIRROR modelling methodology. *J. Theor. Biol.* **113**:311–330.

Hogeweg, P. and B. Hesper, 1991. Evolution as pattern processing: TODO as substrate for evolution. In J.-A. Meyer and S. W. Wilson, editors, *Proceedings of the first international conference on simulation of adaptive behavior on From animals to animats*, pages 492–497. MIT Press, Cambridge, Mass.

Jaeggi, A. V., L. P. Dunkel, M. A. V. Noordwijk, S. A. Wich, A. A. L. Sura, and C. P. V. Schaik, 2010. Social learning of diet and foraging skills by wild immature Bornean orangutans: implications for culture. *Am. J. Primatol.* **72**:62–71.

Matsuzawa, T. and Y. Hasegawa, 1982. Food-aversion conditioning in Japanese monkeys (Macaca fuscata): suppression of key-pressing. *Behav Neural Biol* **36**:298–303.

Matsuzawa, T. and Y. Hasegawa, 1983. Food aversion learning in Japanese monkeys (*Macaca fuscata*). A strategy to avoid a noxious food. *Folia Primatol* **40**:247–255.

Provenza, F. D., 1996. Acquired aversions as the basis for varied diets of ruminants foraging on rangelands. *J. Anim. Sci.* **74**:2010–2020. Diet development.

Smuts, B. B., D. L. Cheney, R. M. Seyfarth, R. W. Wrangham, and T. T. Struhsaker, 1987. Primate Societies. The University of Chicago Press, Chicago.

van de Waal, E., N. Claidière, and A. Whiten, 2014. Wild vervet monkeys copy alternative methods for opening an artificial fruit. *Anim Cogn* .

van de Waal, E. and A. Whiten, 2012. Spontaneous emergence, imitation and spread of alternative foraging techniques among groups of vervet monkeys. *PLoS ONE* **7**:e47008.

van der Post, D. J. and P. Hogeweg, 2006. Resource distributions and diet development by trial-and-error learning. *Behav. Ecol. Sociobiol.* **61**:65–80.

van der Post, D. J. and P. Hogeweg, 2008. Diet traditions and cumulative cultural processes as side-effects of grouping. *Anim. Behav.* **75**:133–144.

van der Post, D. J. and P. Hogeweg, 2009. Cultural inheritance and diversification of diet in variable environments. *Anim. Behav.* **78**:155–166.

van der Post, D. J. and D. Semmann, 2011a. Local orientation and the evolution of foraging: Changes in decision making can eliminate evolutionary trade-offs. *PLoS Comput. Biol.* **7**:e1002186.

van der Post, D. J. and D. Semmann, 2011b. Patch depletion, niche structuring and the evolution of cooperative foraging. *BMC Evol. Biol.* **11**:335.

van der Post, D. J., B. Ursem, and P. Hogeweg, 2009. Resource distributions affect social learning on multiple timescales. *Behav. Ecol. Sociobiol.* **63**:1643–1658.

van der Post, D. J., R. Verbrugge, and C. K. Hemelrijk, 2015. The evolution of different forms of sociality: behavioral mechanisms and eco-evolutionary feedback. *PLoS ONE* **10**:e0117027.

Visalberghi, E. and E. Addessi, 2003. Food for thought: social learning about food in capuchin monkeys, in: D.M. Fragaszy and S. Perry (eds) The Biology of Traditons, pages 187–212. Cambridge University Press.

Visalberghi, E., G. Sabbatini, M. Stammati, and E. Addessi, 2003. Preferences towards novel foods in Cebus apella: the role of nutrients and social influences. *Physiology and Behavior* **80**:341–349. dio:10.1016/j.physbeh.2003.08.004.
